# Progress in detection of and correction for low-energy contamination

**DOI:** 10.1107/S1600576723004764

**Published:** 2023-07-25

**Authors:** Slawomir Domagala, Petrick Nourd, Kay Diederichs, Julian Henn

**Affiliations:** a DataQ Intelligence, Fichtelgebirgsstrasse 66, 95448 Bayreuth, Germany; b Codivert, Żubrowa 15, 01-978 Warsaw, Poland; c Department of Biology, University of Konstanz, Universitätsstrasse 19, 78457 Konstanz, Germany; The University of Western Australia, Australia

**Keywords:** data quality metrics, systematic errors, flawed standard uncertainties, robust metrics, low-energy contamination

## Abstract

Contamination with low-energy radiation leads to an increased number of weighted residuals being larger in absolute terms than three standard uncertainties. For a Gaussian distribution, these rare events occur only in 0.27% of all cases, which is a small number for small- to medium-sized data sets. The correct detection of rare events – and an adequate correction procedure – thus relies crucially on correct standard uncertainties, which are often not available [Henn (2019), *Crystallogr. Rev.* **25**, 83–156]. It is therefore advisable to use additional, more robust, metrics to complement the established ones. These metrics are developed here and applied to reference data sets from two different publications about low-energy contamination. Other systematic errors were found in the reference data sets. These errors compromise the correction procedures and may lead to under- or overcompensation. This can be demonstrated clearly with the new metrics. Empirical correction procedures generally may be compromised or bound to fail in the presence of other systematic errors. The following systematic errors, which were found in the reference data sets, need to be corrected for prior to application of the low-energy contamination correction procedure: signals of 2λ contamination, extinction, disorder, twinning, and too-large or too-low standard uncertainties (this list may not be complete). All five reference data sets of one publication show a common resolution-dependent systematic error of unknown origin. How this affects the correction procedure can be stated only after elimination of this error. The methodological improvements are verified with data published by other authors.

## Abstract 2

A new robust indicator for low-energy contamination is presented and applied to experimental data. The standard correction procedure for low-energy contamination is improved.

## 1. Introduction

The identification, description and removal of systematic errors in diffraction experiments has become increasingly important in recent years as, with high redundancies, the statistical error has been reduced to such an extent that sytematic errors are now the dominant errors in diffraction data. Consequently, there is an increased need for the detection and quantification of systematic errors, even in small-molecule data sets. A well understood systematic error is contamination with low-energy radiation like 3λ contamination: radiation with triple the basic wavelength λ emerges due to imperfect monochromaticity. The low-energy contribution is diffracted in the same way as the basic wavelength but at tripled Miller indices *h*, *k*, *l*.

Low-energy contamination appears when Mo radiation is combined with mirror optics, as in this combination the reflection angles in the mirror optics support total reflection of wavelengths with double and triple the basic wavelength which are found in the continuous emission spectrum. Cases of 3λ contamination have been discussed in the literature (Macchi *et al.*, 2011 ▸; Krause *et al.*, 2015 ▸), whereas 2λ contamination has not yet been reported. Low-energy contamination is not expected for Cu and Ag radiation or synchrotron radiation, or for Mo radiation experiments in combination with other devices like a traditional graphite monochromator. The presence of 3λ radiation can be avoided physically by blocking the low-energy radiation with thin foils, but if this opportunity is not taken in the experiment, low-energy contamination can still be corrected for. Correction procedures always have their own advantages and disadvantages, and it is of course advisable to avoid errors in the first place.

Krause *et al.* (2015 ▸) proposed a correction procedure in which the systematic difference between unaffected (by low-energy contamination) and affected intensities is fitted by a weighted least-squares procedure: 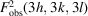 = 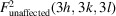 + 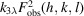 . The fit parameter *k*_3λ_ quantifies the degree of 3λ contamination and is used for a correction procedure,

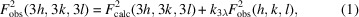

in which the unknown entity 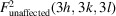 is replaced by the known entity 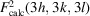 .

It can be expected that this approximation will hold better the lower the overall contamination with systematic errors is. Conversely, this approximation is invalidated in the presence of many or strong systematic errors, as the calculated intensities are more and more biased by the increasing errors.

Another correction procedure proposed by Krause *et al.* (2015 ▸) treats 3λ contamination as a (de)twinning problem with a virtual twin domain, leading to similar results. In the present work the focus is on the first correction procedure as indicated by equation (1).

## 2. Traces of 3λ contamination

Established traces of 3λ contamination are radially smeared out reflection spots in the frames and a transfer of intensity from base reflections *h* *k* *l* to corresponding reflections 3*h* 3*k* 3*l*. A consequence of this process is that specifically reflections with Miller indices which are all multiples of three (or with zero index), like 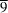 3 18, 300 or 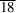 9 0, contribute strongly, disproportionately to their relative share, to the number of large weighted residuals |Δ/σ(*I*_o_)| = |ζ| > 3, provided that the σ(*I*_o_) are absolutely and relatively correct,^1^ and maybe also provided that no other strong systematic error is present, as this may obstruct or counteract the traces of 3λ contamination in the residuals. It may therefore be a good idea to work out more consequences of low-energy contamination in order to establish a fingerprint that does not rely on just one criterion like the over-representation of reflections with Miller indices being multiples of two or three in the subset of large residuals. For the sake of simplicity, reflections with all Miller indices being multiples of three are abbreviated by the symbol *m*_3_. In an analogous way, reflections with all Miller indices being multiples of two are abbreviated by *m*_2_.

In total, one would expect, as a consequence of this specific systematic error, the following characteristics:

(1) A significant contribution of *m*_3_ to rare events |ζ| > 3 [provided the σ(*I*_o_) are correct].

(2) A large contribution of *m*_3_ to all strong residuals [not only to those with ζ > 3, independent of absolutely correct σ(*I*_o_)].

(3) An increased number of positive weighted residuals #ζ_+_ and a decreased number of negative weighted residuals #ζ_−_. Whether the number of positive residuals in excess is significant can be quantified by dividing the difference by the square root of the number of all residuals, (#ζ_+_ − #ζ_−_)/(*N*_obs_)^1/2^. This corresponds to the significance of the deviation from zero in a random-walk process, where positive and negative steps have the same probability 0.5.

(4) A positive shift of the mean value of all residuals 〈ζ〉. Instead of looking only at the mean value of the residuals, it is more precise to monitor the significance of the deviation of the mean value of the residuals from zero: 〈ζ〉/σ(〈ζ〉) with σ(〈ζ〉) = [var(ζ)/*N*_obs_]^1/2^.

(5) On average, stronger positive weighted residuals compared with negative ones, 〈ζ_+_〉 > 〈|ζ_−_|〉.

(6) More strong positive outliers than negative ones, 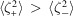 .

(7) An increase in *wR*(*F* ^2^).

(8) An increase in goodness of fit (GoF).

The above points are not all independent of each other. Descriptor (1) is a standard indicator which in this work is additionally endowed with an error bar. Descriptor (2) is a new and more robust indicator based on histograms and ranking rather than absolute numbers, which will be explained in greater detail below. Descriptors (3) to (5) are just more specific consequences of 3λ contamination and would apply to 2λ contamination in an analogous way. Normal probability plots (Abrahams & Keve, 1971 ▸) would clearly display points (3)–(6) by showing *more* and *stronger* outliers on the right-hand side, as well as a slightly right-shifted value at zero.

In consequence, a successful correction for 3λ contamination reverses all the above-listed signs (1)–(8). If the effects are not reversed, then other systematic errors are necessarily present, the causes of which may or may not be known.

## 3. New detection metrics for low-energy contamination

**Figure 1:**
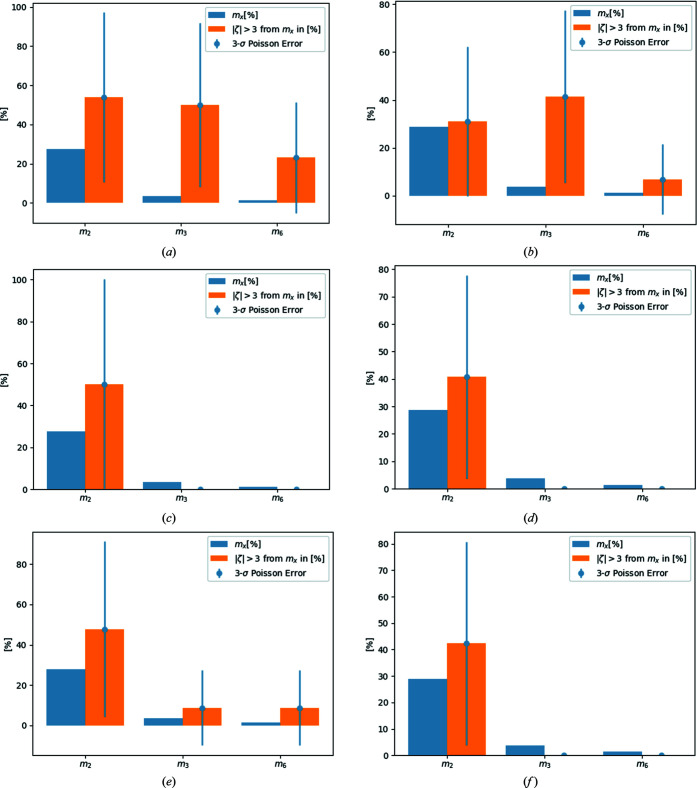
Plots of 2λ and 3λ contamination and twinning. The left-hand column shows data set **1** and the right-hand column shows data set **2**. (Left) The 3λ correction procedure reduces the contribution of multiples of three to the rare events, as indicated by the orange bar in the middle of panel (*a*) compared with panel (*c*). The contribution from multiples of two, however, is insignificant in (*a*) and remains insignificant for the corrected and filtered data sets, as indicated by the left-hand orange bar in panels (*a*), (*c*) and (*e*). In data set **2** (right-hand column) the 3λ correction procedure also reduces the contribution of multiples of three to the rare events, but the initially insignificant contribution of multiples of two *increases* to a level where it may just become significant. This is also the case in the filtered data set.

In order to make the existing metrics additionally more significant and impactful, it is suggested that:

(i) Multiples of two, three and six be always monitored together.

(ii) An error bar be added to the percentage of multiples based on Poisson statistics. This allows a judgement on whether a deviation of the existing value from the expected value is significant or insignificant. As an example, look at Fig. 1(*a*) below[Sec sec4], where a 3σ error bar is added to the rare events from multiples of two, *m*_2_, to the rare events from multiples of three, *m*_3_, and to the rare events from multiples of six, *m*_6_. Only the 3λ contamination signal is significant.

(iii) The important items from the list (1)–(8) above be cross-checked for evaluation of the initial state of the data set and the progress of the correction process. Which of the items are important is investigated later.

### 3.1. Error bar based on Poisson statistics for comparing the share of rare events from multiples with the share of multiples from all reflections

A standard technique to detect low-energy contamination is to compare the share of rare events from multiples with the share of multiples from all reflections. These shares should be equal within statistical fluctuations. If, for example, 3.54% of all reflections *N*_obs_ = 1697 are multiples of three, then these should contribute to approximately 3.54% of all rare events |ζ| > 3. But how large are the statistical fluctuations? They are now specified by an error bar derived from Poisson statistics. For example, if there are in total 26 rare events |ζ| > 3, and 13 rare events are from multiples of three, the 1σ error bar is ±13^1/2^ = ±3.61 and the share of rare events from multiples of three to all rare events is 50% (13/26). This is much higher than the expected 3.54%. But the absolute numbers of rare events are also quite small, so it is not yet clear whether 50% of the share could still be within statistical fluctuations or not. This is decided by the error bar, which is calculated as (3 × 13^1/2^)/26 = 0.42, *i.e.* approximately 42%. A lower bound of the statistical fluctuations is therefore 50% − 42% = 8%, which is still larger than 3.54%. The contribution of *m*_3_ to all rare events is therefore too large to be consistent with statistical fluctuations. The numbers discussed here are from data set **1_uncorr** (see Section 4) and are visualized in the middle part of Fig. 1(*a*), where the blue bar represents the fraction *m*_3_ of all reflections (3.54%), the orange bar represents the contribution of *m*_3_ to all rare events (50%) and the error bar is given by ±42%. Some of the discussed numbers are also given in Table 2 below.

The error bar is helpful to evaluate whether the expected (3.54%) and found (50%) shares are possibly just within statistical fluctuations. In this publication we adopt the convention to use a 3σ event as the criterion, *i.e.* if the result deviates by three standard deviations or more, this is assumed to be significant. There is no rigorous proof for this assumption; it is simply based on convention. For the numbers just discussed, the above-mentioned 42% represents a 3σ event. The expected 3.54% deviates from 50% by more than 3σ.

### 3.2. Histograms of multiples in equal bins of weighted residuals

It is known that the σ(*I*_o_) are often inadequate [see *e.g.* Henn (2019 ▸)] as they are designed specifically for the purpose of making the weighted residuals independent of the resolution, with the help of the weighting scheme parameters. In order to construct metrics that are less dependent on the correct σ(*I*_o_), it is suggested to use histograms of the multiples in five or ten bins of the residuals or in an appropriately chosen number of bins. Instead of analysing the largest residuals |ζ| > 3 exclusively, like above, *all* residuals are analysed and the analysis is no longer based on the numeric value of the residual, which depends on the correct σ(*I*_o_). The analysis is further based on the ranking of residuals ζ, rather than the ranking of absolute values |ζ| of the residuals. This is worth mentioning, because with low-energy contamination it is expected that specifically just the number of strong positive residuals will increase, but not the number of strong negative residuals. If by some exotic error just the negative residual of, say, *m*_3_ were to become larger in absolute terms, this would be falsely attributed to 3λ contribution if the analysis were based on |ζ| rather than ζ.

## 4. Application of the new metrics

The new metrics are applied to data sets known to be contaminated by 3λ radiation, used in the study reported by Krause *et al.* (2015 ▸) and described in greater detail there. A very brief characterization is given in Table 1 ▸. Each of these five data sets, herein numbered **1**–**5**, exists in three different forms: the ‘uncorrected’ (for low-energy contamination) form, the ‘corrected’ form, where the correction procedure as described by Krause *et al.* (2015 ▸) is applied for an *a posteriori* correction of the experimental data, and the ‘filtered’ form, in which a thin Al foil was used during data collection to block the low-energy radiation physically.

### 4.1. Application of the error bar in low-energy contamination

As an example, reference data sets **1** and **2** are now discussed in greater detail. The results for the other reference data sets are also briefly given. Fig. 1 ▸ displays information for multiples of two, three and six for reference data set **1** (left-hand column) and reference data set **2** (right-hand column). The situation prior to the correction procedure is depicted in the first row for each data set, the situation after application of the correction procedure is shown in the second row, and the ‘filtered’ data sets, where a thin metal foil physically blocked the low-energy radiation, are shown in the third row. For each data set (**1** and **2**) and for each state [uncorrected (suffix **_uncorr**), corrected (**_corr**) and filtered (**_filter**)] the percentage fractions of multiples of two, three and six are given as blue bars. The corresponding fractions of rare events from the multiples of two, three and six to all rare events are given as orange bars next to the blue ones. A 3σ error bar based on Poission statistics is attached to these.

Fig. 1 ▸(*a*) shows that initially there are significant (3.35σ, based on Poisson statistics) contributions from *m*_3_, but not from *m*_2_ (1.83σ) or *m*_6_ (2.32σ), to the rare events in data set **1_uncorr**. After application of the correction process [Fig. 1 ▸(*c*)], the contributions from *m*_3_ and *m*_6_ vanish completely, whereas the contribution of *m*_2_ remains insignificant (with 0.98σ).

In reference data set **2**, there is also initially a 3.15σ significant signal for 3λ contamination [Fig. 1 ▸(*b*)]. In the corrected data set, the signal for 3λ contamination is insignificant [Fig. 1 ▸(*d*)]. Reference data set **3** shows a very significant (4.32σ) 3λ contamination signal that becomes insignificant after application of the correction procedure (1.69σ) and for the filtered data set (0.78σ; for the corresponding plots see the supporting information). In reference data set **4**, the initially significant 3λ signal (3.79σ) is also insignificant (0.38σ) after correction and for the filtered data set (0.42σ). Only in reference data set **5** is the signal for 3λ correction initially not significant (2.14σ) according to a 3σ criterion. However, for the corrected data set (0.11σ) and for the filtered data set (0.04σ) the signal becomes even more insignificant than for data sets **1**–**4**.

As all examples are known to be contaminated by 3λ radiation, the findings for data set **5** raise the question of why the contamination remains insignificant in this data set. This is discussed below in greater detail, but for the moment it needs to be kept in mind that signals with a significance less than 3σ might still reveal low-energy contamination.

### 4.2. Application of the histograms of multiples in bins of increasing residuals

**Figure 2:**
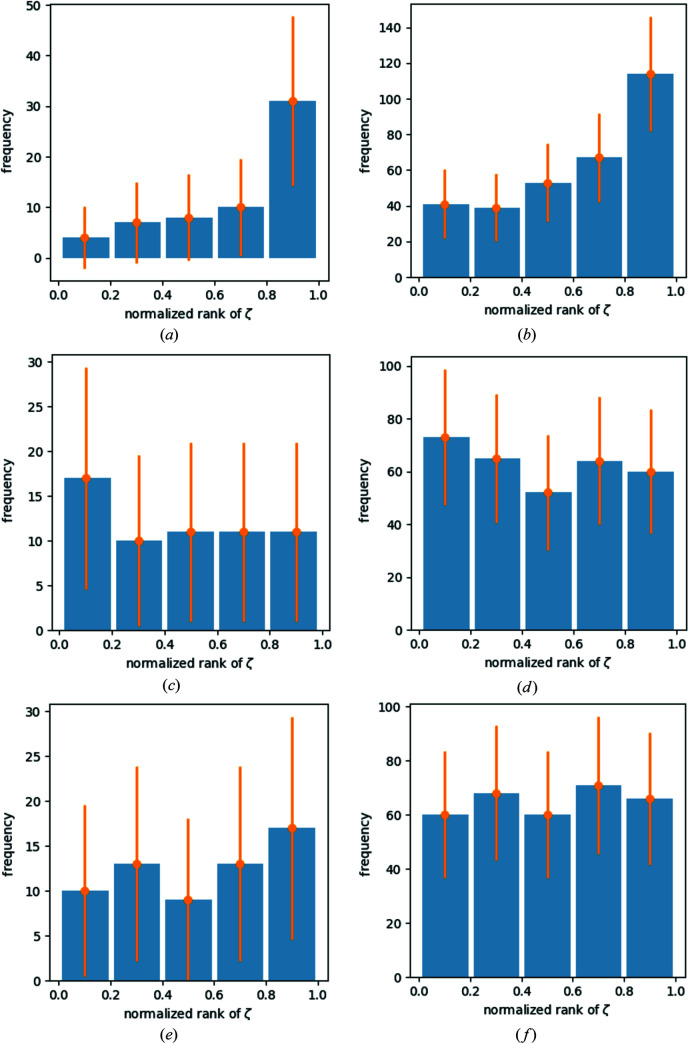
The left-hand column shows data set **1** and the right-hand column shows data set **4**. Histograms of multiples of three, *m*_3_, in approximately equally populated bins of the weighted residuals ζ in increasing order (on the left the most negative, on the right the most positive residuals). The respective first bin gives the integer number *n*_1_ of *m*_3_ for the lowest (most negative) 20% of residuals, while the respective last bin gives the integer number *n*_5_ of *m*_3_ for the largest (most positive) residuals. The error bars mark 3σ and are ±(*n*_*i*_)^1/2^ according to Poisson statistics. (Left) Initially the *m*_3_ are polarized towards positive residuals. (*a*) The more positive the residual, the more *m*_3_ are in the respective bin. (*c*) The correction procedure overcompensates the 3λ effect: after application of the correction procedure most multiples of three are found in the bin with the most negative residuals. (*e*) In data set **1_filter**, the multiples of three are approximately equally distributed, with a statistically insignificant tendency to find more *m*_3_ again for the largest positive residuals (largest bin on the far right). Data set **4** initially also shows a polarization of *m*_3_ to positive weighted residuals (*b*), but after correction (*d*) shows a uniform distribution of *m*_3_ with respect to the weighted residuals, like for the filtered data set **4** (*f*) and in contrast to (*b*).

As an example, data sets **1** and **4** are discussed. The corresponding histograms can be found in Fig. 2 ▸. Each bar in all histograms is annotated with a Poisson-based 3σ error bar. The left-hand column describes data set **1** and the right-hand column data set **4**. Figs. 2 ▸(*a*) and 2 ▸(*b*) show that low-energy contamination leads to an overall polarization of the residuals with respect to occurrences of *m*_3_ in data sets **1_uncorr** and **4_uncorr**: the more positive the residual, the more appearances of *m*_3_. The most negative 20% of residuals show a low number of *m*_3_, with a tendency to increase for the next 20% of residuals and so on up to the 20% most positive residuals with significantly more *m*_3_ than in all other bins. After the correction procedure, suddenly the 20% most *negative* residuals display the largest population with *m*_3_ in data set **1_corr** [Fig. 2 ▸(*c*)]. This is interpreted as an overcompensation process.

The data set **1_filter** again shows a slight but insignificant tendency to a polarization of *m*_3_ to positive residuals, while in data set **4_filter** there is no such tendency visible [Fig. 2 ▸(*f*)]. The histogram in reference data set **4_corr** shows a successful correction procedure.

In reference data set **3_uncorr** there is initially a peak contribution of *m*_3_ to the 20% of largest (positive) residuals visible. The corrected data set indicates that the most negative 20% of residuals show an increased frequency of contributions from *m*_3_ in the corresponding histogram. Using ten bins instead of five reveals that the most positive 10% also show an increased number of *m*_3_ reflections. Both signals are just on the verge of becoming significant. The interpretation of this pattern is not clear. If the remaining contributions to the most positive signals are interpreted as an undercompensation process and additional contributions of *m*_3_ are interpreted as an overcompensation process, then in this data set there would be simultaneously signs of both under- and overcompensation, which seems to be inconsistent. As long as the correct interpretation for this signal is not found, it is marked as showing simultaneously signs of over- and undercorrection. Due to the small peak for the residuals close to zero in the bins in the middle of the plot, there is also a resemblance to reference data set **2_corr**. In reference data set **5**, the histograms prior to correction also show peak distributions of *m*_3_, specifically for the largest (most positive) residuals. After correction there are no distinct signs of remaining errors. There are, however, two interesting and unexplained features: The negative residuals tend to show in total fewer reflections of *m*_3_ compared with the positive residuals. This is particularly clearly shown when using ten bins instead of five. The distribution should be uniform. The reference data set **5_filter** shows a strong polarization of the *m*_3_ reflections with respect to the residuals: the more positive the residuals, the larger the fraction of *m*_3_. This kind of distribution is expected and consistently observed for all uncorrected reference data sets (**1_uncorr**, **2_uncorr**, **3_uncorr**, **4_uncorr** and **5_uncorr**) and they all display a corresponding 3λ contamination signal. In this case the polarized residuals occur for the filtered data set **5_filter** and it does *not* display a significant signal for *m*_2_, *m*_3_ or *m*_6_. This remains a riddle at this point in the discussion.

### 4.3. *A priori* expectations of low-energy contamination

Apart from the discussed new metrics, there are the *a priori* expectations for the signs of low-energy contamination (3)–(8) as introduced above. Discussing these in detail may give hints for successful and unsuccessful correction procedures and for other systematic errors that may interact with the correction procedure. A first hint of interactions was found with an increased, albeit insignificant, 2λ contamination signal in reference data set **1** that may interact with the application of a 3λ correction procedure.

## 5. The descriptors in detail

### 5.1. Rare events from *m*_3_ and contribution from *m*_3_ to 20% of the largest residuals

In all cases, and as expected, the 3λ correction procedure reduces (i) the total number of rare events, (ii) the relative contribution of *m*_3_ to those rare events and (iii) the contribution from *m*_3_ to the 20% of largest residuals. The reduction in rare events is, however, only very small in some cases, for example in data set **4**, where in the uncorrected data set 19 out of a total of 69 rare events are from *m*_3_ (27.54%, see second column in Table 2 ▸) and after correction only three rare events are from *m*_3_. However, the total number of rare events (65) remains close to the initial value of 69. This may be a hint that low-energy contamination is not the dominant systematic error in data set **4**.

### 5.2. Shift in the significance of the mean of the residuals, as given by 〈ζ〉/σ(〈ζ〉)

In all cases, and as expected, the significance of the deviation of the mean residuals from zero decreases when 3λ correction procedures are applied. It is important to note, however, that some mean values are different from zero with high significance before *and* after the correction, as in the case of data set **3** (before/after: 5.47/4.03) and data set **4** (6.40/5.37). This is evidence of systematic errors in itself, as a significant deviation from zero indicates non-random contributions to the mean value, *i.e.* contamination with systematic errors. The question of which types of systematic error lead to such large deviations is an open research topic and cannot be answered fully in this work, but it will be touched on again below. It is an important question, though, as significantly large absolute deviations of the mean value of the weighted residuals from zero larger than three are widespread. In the 127 data sets published by *IUCrData* (https://iucrdata.iucr.org/) and discussed by Henn (2019 ▸) ▸, they appeared in 66 (52%) cases, but remained unmentioned – and most likely undetected – in all publications where they appeared. On average, the absolute significance of the deviation of residuals from zero for the 127 data sets was 4.30.

### 5.3. Number of positive and negative residuals as given by (#ζ_+_ − #ζ_−_)/(*N*_obs_)^1/2^

As pointed out above, low-energy contamination leads to an increase in, specifically, the positive residuals. Consequently, the correction procedure should reduce the number of positive residuals further. However, the number of positive residuals *increases* after correction for reference data sets **1**, **2**, **3** and **5**, as can be seen from Table 2 ▸, where the significance of the difference between the number of positive and negative residuals, (#ζ_+_ − #ζ_−_)/(*N*_obs_)^1/2^, is given in the fifth column. This is clearly counterintuitive and needs an explanation. The obvious explanation is that there are additional systematic errors in these data sets. Only in data set **4** does the number of positive residuals decrease after correction. Data sets **3** and **4** (including the filtered data sets) show a significant excess of positive residuals.

### 5.4. Mean of positive and negative residuals

In order to track the effect of the correction procedure, it is also helpful to monitor the mean values of the positive and negative weighted residuals separately, as 3λ contamination is expected to lead selectively to stronger positive residuals only. In general, positive and negative residuals should show the same average value when no systematic errors apply. In the case of a Gaussian distribution, this expectation value is given by 〈|ζ_±_|〉 = (2/π)^1/2^α with 0 < α = (*N*_obs_ − *N*_par_)/*N*_obs_ < 1. For the separated samples of positive and negative residuals, the standard deviation of their respective mean values is calculated from their respective variances by

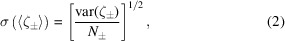

where 

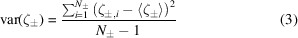

indicates the sample population variance of either the positive or the negative residuals and *N*_±_ indicates either the number of positive or the number of negative residuals. The error bar as given in equation (2) enables control of the consistency of the separate mean values from the positive and negative residuals and additionally of their consistency with the expectation value of a Gaussian distribution. The mean values of the positive and absolute negative residuals are given together with a 3σ error in columns seven and eight of Table 2 ▸.

*Positive residuals 〈ζ_+_〉.* The mean values 〈ζ_+_〉 tend to be slightly too large, but are surprisingly often in accordance with the expectation value from a Gaussian distribution (2/π)^1/2^α. The positive residuals tend to be larger than their reference value before and after 3λ correction. The correction procedure only leads to a decreasing mean value of the positive residuals 〈ζ_+_〉 in the case of data sets **3** and **5**, while in the other cases it remains the same within the given digits, or even *increases*, as for data set **2** (from 0.76 to 0.78, see Table 2 ▸, column 8). An increase is clearly counterintuitive. A possible explanation is an *error compensation* process: removal of the 3λ error leads to visibility of other errors, which were obstructed or counteracted by the former.

*Negative residuals 〈|ζ_−_|〉.* The correction procedure leads to an increasing mean value of the absolute negative residuals 〈|ζ_−_|〉 in all cases, and also in the individual case of set **5**, where the mean value was too large from the start (〈|ζ_−_|〉 before/after/reference: 0.77/0.79/0.75). In all cases where the correction was applied, the mean value of the positive residuals is larger than the mean value of the absolute negative residuals prior to and after the correction procedure. The mean value of the absolute negative residuals for the uncorrected data sets **1**, **2**, **3** and **4** is *lower* than the reference value. This may be interpreted as a hint that the standard deviations are too large in these sets in general, with an additional error that increases the positive residuals selectively, or it may be connected to a shift of the residual distribution as a whole to positive values. Both cases may result in a shift of the residuals to positive values, as just discussed in Sections 5.2 and 5.3. A positive shift of any symmetric residual distribution would selectively lead to increased frequency and strength of positive residuals compared with the negative ones.

### 5.5. Mean of positive and negative squared residuals

The mean value of the *squared* residuals emphasizes outliers. As the *m*_3_ observed intensities are increased by 3λ contamination by Δ_3λ_ ≥ 0, it is expected that more large positive residuals ζ > 3 will be found for these, which implies that negative outliers ζ < −3 from *m*_3_ are reduced. It is consequently expected that the mean of the squared positive residuals will be significantly larger than its reference value and that the correction procedure will lead to a reduction in the mean value of the squared positive residuals. For the mean value of the negative squared residuals it is expected, prior to the correction, that (i) 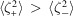 and (ii) 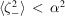 in a data set without any other systematic error. While (i) is observed in all data sets, (ii) is often not the case, which shows that the assumption of low-energy contamination being the sole source of systematic errors is too optimistic. It is also expected that (iii) 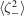 will increase after correction.

*Positive squared residuals 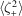 .* In all data sets, the mean squared positive residuals behave as expected, *i.e.* they are (i) larger than the corresponding negative value and (ii) in most cases significantly larger than the reference value α^2^ before correction, and (iii) reduced after correction. The resulting values after correction are, however, *all* above the reference value α^2^. This is also the case for the filtered data sets. The reference value α^2^ corresponds to the case of a Gaussian distribution of residuals without any systematic errors.

*Negative squared residuals 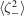 .* The mean squared negative residuals all increase after application of 3λ correction, as expected. Some resulting values are, however, still *smaller* than the reference value (sets **1** and **4**; in **4** with significance). Expectation values that are significantly too small may be a hint of too-large σ(*I*_o_) values. The *primary* cause would be overfitting in this case. On the other hand, it could be an effect from another (as yet unidentified) error that leads to large positive shifts in the mean values of the residuals 〈ζ〉/σ(〈ζ〉). These positive shifts are largest after correction for data sets **1**, **3** and **4**, *i.e.* for exactly those data sets with the lowest mean values 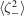 , with 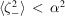 in the case of data sets **1** and **4**. In this case, the significantly reduced mean value of the negative squared residuals may not point directly to the primary cause, but instead may be the effect of an unknown systematic error that leads to positive shifts in the mean values of residuals, *i.e.* a *secondary* effect. As will be discussed later, data set **3** does show (a modelled but maybe incomplete) disorder. As a working hypothesis until validation or falsification, it may be assumed that a significantly reduced mean value of the negative squared residuals may be a secondary effect of disorder, together with a significantly positive shifted mean value of the residuals. Of course, this pilot study cannot answer all relevant questions immediately, and the findings need to be validated or falsified with a larger number of examples over time.

### 5.6. Weighted agreement factors and GoF

The weighted agreement factors all decrease after application, if sometimes only slightly. The significance of the changes was tested with the Hamilton test (Hamilton, 1965 ▸). They are all significant at the significance level 0.005.

The GoF also decreases or remains virtually unaffected, as for reference data set **2**. There, the reduction in 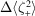 = −0.25 due to the correction procedure is compensated by a corresponding increase 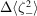 = 0.25. As a result, the GoF remains constant.

### 5.7. Normal probability plots

**Figure 3:**
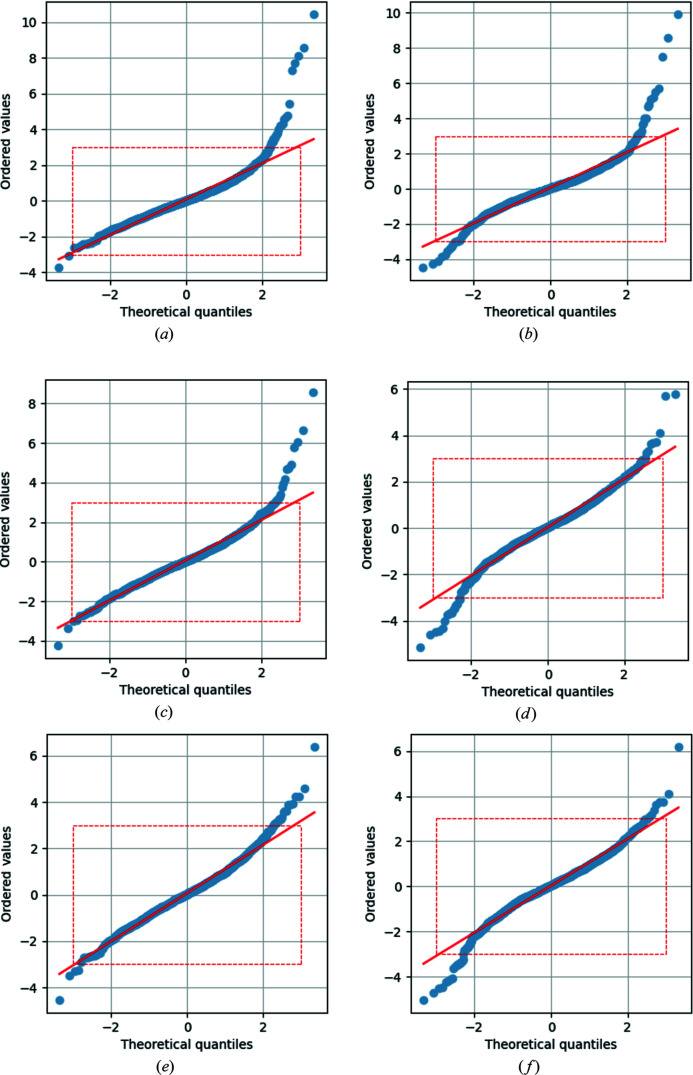
Probability plots. The left-hand column shows data set **1** and the right-hand column shows data set **2**. (Left) The 3λ correction procedure reduces the number and strength of rare events ζ > 3, as indicated by the number and strength of outliers in the right periphery of panel (*a*) compared with (*c*). They are, however, not eliminated and are also visible in the filtered data set shown in panel (*e*). Changes in the left-hand periphery are much smaller. The observations are similar in data set **2** (right-hand column).

The normal probability plot (NPP) is a valuable diagnostic tool in general, and in particular in the case of 3λ contamination. It shows the above-mentioned characteristic features of stronger and more frequent positive residuals compared with the negative ones. Note that in the case of modifying the expected distribution to *e.g.* the *t* distribution, as proposed by Hooft *et al.* (2009 ▸), in order to accommodate the expected outliers, they might not be visible as such any more. Instead of modifying the expected distribution, we propose to stay with the normal distribution and investigate deviations from it in order to describe, identify and ultimately remove systematic errors.^2^ As an example, the correction procedure for reference sets **1** and **2** is discussed in greater detail in this section and depicted in Fig. 3 ▸. The findings are similar for the other reference data sets. The NPPs for all data sets can be found in the supporting information. The number and strength of positive outliers is reduced by the correction process, as can be seen by comparing Fig. 3 ▸(*a*) with Fig. 3 ▸(*c*) for reference data set **1** and Fig. 3 ▸(*b*) with Fig. 3 ▸(*d*) for reference data set **2**. Large positive outliers remain in both cases and are only visible when the full range of the NPP is shown (not limited to a region between −3 and 3). The left-hand sides of the NPPs show comparably few changes.

### 5.8. Problems with σ(*I*_o_)

When the σ(*I*_o_) = [s.u.^2^ + (*aP*)^2^ + *bP*]^1/2^, with *P* = *fI*_o_ + (1 − *f*)*I*_c_, are (distinctly) too small, this is easily detected, *e.g.* in Bayesian conditional probability (BayCoN) plots [see, for example, Williams *et al.* (2019 ▸) and Henn & Meindl (2014*b* ▸)]. When, in contrast, the σ(*I*_o_) are too large, other systematic errors can be disguised, as this leads to more uniform BayCoN plots and artificially lowered GoF values.

Standard deviations that are too large or too small have the potential to invalidate the results from the least-squares procedure and affect many metrics [like GoF and *wR*(*F* ^2^)] used to judge the process. Additionally, they play a role in the correction procedure when they are used as weights and may lead to over- or undercompensation.

A particularly helpful metric for the detection of too-large standard deviations is the alternative goodness of fit (aGoF), as this might become smaller than one in this case, whereas the GoF may still remain larger than one (Henn, 2019 ▸, 2016 ▸). To give a very brief explanation for these findings: The deviation of the GoF is based on the χ^2^ distribution that describes independent identically distributed random numbers. In many, if not most, published data sets, the weighted residuals are not random numbers, as can easily be verified for example by highly significant correlation coefficients between the squared weighted residuals and, for example, σ^2^(*I*_o_). In 127 analysed highlighted data sets published in *IUCrData*, 28 (22%) showed a corresponding large correlation coefficient with significance larger than three and the average significance of the correlation coefficient cc[ζ^2^, σ^2^(*I*_o_)] for all 127 data sets was 3.21 (data not published). The weighting scheme may play an important role in this.

**Figure 4:**
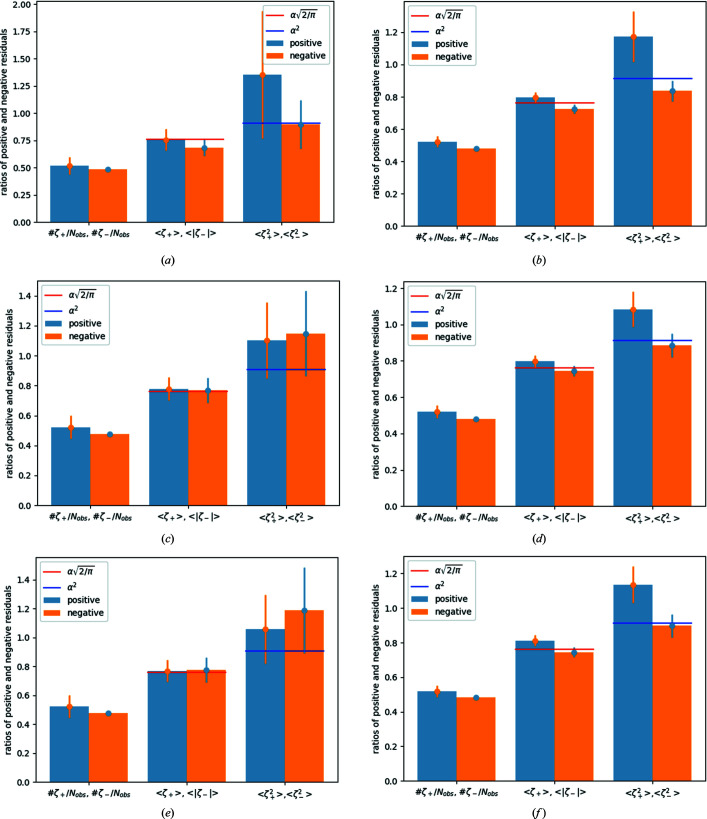
The left-hand column shows data set **2** and the right-hand column shows data set **4**. The blue bars refer to positive weighted residuals and the orange bars to negative. The first pair of bars in each plot displays the fraction of positive and negative residuals. A 3σ error bar is attached to the positive values, which indicates the range of statistical fluctuations according to a random-walk process with the same probability for positive and negative steps. The pair of bars in the middle of each plot display the mean values of the positive residuals and of the absolute value of the negative residuals. These mean values should be consistent within statistical fluctuations. The range of statistical fluctuations is given by the error bars. Additionally, a reference value (red horizontal line) is given. When the residuals are Gaussian distributed, the mean values should both be consistent with this reference value. The last pair of bars in each plot display the mean values of the squared positive residuals and of the squared negative residuals, together with their respective 3σ error bars and the reference value for a Gaussian distribution (blue horizontal line). The squaring emphasizes outliers.

Table 2 ▸ shows that aGoF ≤ 1 for reference data sets **2** and **4** (and **3_filter**), *i.e.* overfitting applies in these sets. This is attributed to too-large standard deviations and confirmed by the mean values of the (squared) positive and negative residuals: the mean values 〈ζ_−_〉 and 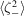 all remain *below* the reference value in reference data set **4** [see Table 2 ▸ and Figs. 4 ▸(*b*), 4 ▸(*d*) and 4 ▸(*f*)]. A similar, but not as distinct, tendency is visible in data set **2**, where 〈ζ_+_〉 is in accordance with the reference value despite known low-energy contamination. It should be larger than the reference value for the contaminated data set. After correction and for the filtered data set, the mean values 〈ζ_±_〉 are in accordance with their reference values, but there are systematic errors remaining in all data sets, *i.e.* the resulting mean values should be larger than the reference value [see Table 2 ▸ and Figs. 4 ▸(*a*), 4 ▸(*c*) and 4 ▸(*e*)].

The aGoF shows comparably large values for all reference data sets **5** (**5_uncorr**, **5_corr** and **5_filter**). This is a hint of unidentified strong systematic errors in this data set. One possible systematic error leading to high values of the aGoF is σ(*I*_o_) values that are too small. Underestimation of σ(*I*_o_) leads in some very distinct cases to non-uniform BayCoN plots [ζ^2^, σ(*I*_o_)], which is not the case for data set **5** {χ^2^[ζ^2^, σ(*I*_o_)] = 109.88, 118.59 and 120.46 for uncorrected, corrected and filtered data, respectively}. Values of σ(*I*_o_) that are much too small are thus excluded as a possible cause for the large aGoF in all members of data set **5**. There are, however, two very large outliers in the scatter plots *I*_o_ versus *I*_c_ for the strongest reflections in these data sets that might point to extinction, detector saturation or partial shadowing. These two reflections show the largest Δ values, which may significantly increase the aGoF.

## 6. Discussion

The expected specific features can now be categorized into ‘robust’ and ‘fragile’ traces of low-energy contamination. The robust ones also show up in the presence of other systematic errors, while the fragile ones are easily obstructed or counter­acted by other systematic errors.

### 6.1. Expected features after 3λ correction visible in almost all sets despite the presence of other errors

(i) Shift to lower values of the significance of deviation of the residuals from zero, 〈ζ〉/σ(〈ζ〉).

(ii) Total reduction of 〈ζ^2^〉, leading to lower agreement factors and GoF (exceptions are **2_uncorr** and **2_corr**). Note that this is expected due to the new degree of freedom introduced; *i.e.* a reduction is *per se* not a confirmation of the correctness of the procedure.

(iii) Reduction in 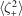 (not statistically significant).

(iv) Increase in 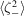 (significant for data sets **1**, **2** and **3** when comparing uncorrected and corrected data sets).

### 6.2. Expected, but not visible, features after 3λ correction

(i) Reduction in the number of positive residuals in the corrected data sets compared with the contaminated data sets. In all data sets except **4_corr** the number of positive residuals increases after correction, as can be seen from the increased significance of the positive excess residuals (#ζ_+_ − #ζ_−_)/(*N*_obs_)^1/2^.

(ii) Reduction of aGoF = [〈Δ^2^〉/α〈σ^2^(*I*_o_)〉]^1/2^. For data sets **1**, **2** and **4**, aGoF *increases* after correction. This reflects an increase in the mean unweighted residuals 〈Δ^2^〉 compared with 〈σ^2^(*I*_o_)〉. As an example, the ratios 〈Δ^2^〉/〈σ^2^(*I*_o_)〉 prior to and after correction for data set **1** are 1.02 (**1_uncorr**) and 1.19 (**1_corr**), for data set **2** they are 0.78 (**2_uncorr**) and 0.92 (**2_corr**), and for data set **4** they are 0.60 (**4_uncorr**) and 0.71 (**4_corr**). When comparing the weighted agreement factors for two sets there is the problem that not only 〈Δ^2^〉 changes but also 〈σ^2^(*I*_o_)〉, due to changing weighting scheme parameters. The weighted agreement factor just gives the total change. Looking at the aGoF, the total change can be broken down to a change in the mean of unweighted squared residuals and the mean of squared standard deviations of the observed intensities. As an example, in data set **1_uncorr** 〈Δ^2^〉 = 2.89 × 10^4^ and 〈σ^2^(*I*_o_)〉 = 2.83 × 10^4^, and in **1_corr** 〈Δ^2^〉 = 2.53 × 10^4^ and 〈σ^2^(*I*_o_)〉 = 2.14 × 10^4^, *i.e.* the correction procedure leads to a substantial decrease in 〈Δ^2^〉 to 88% of the starting value, although, due to changes in the weighting scheme parameters, 〈σ^2^(*I*_o_)〉 decreases even more to 76% of the starting value. In total that leads to an increase in the aGoF and to a slight decrease in the GoF. Application of the weighting scheme actually hampers direct comparison between the agreement factors and GoF values from different refinements.

The increase in aGoF after correction is interpreted as a loss of error compensation after the application of the low-energy correction, as the aGoF would clearly decrease otherwise. As just mentioned, this increase is related to changes in the weighting scheme parameters, which would all be zero in the case of correct s.u.(*I*_o_). In data set **1_uncorr** *b* = 2.3981 and in data set **1_corr** *b* = 1.9011, *i.e.* both are large and indicate by and in themselves the presence of systematic errors. It is again an ‘elephant in the room’ situation when low-energy contamination correction leads to a comparatively small decrease in the weighting scheme parameter from *b* = 2.3981 to *b* = 1.9011, but the question of why *b* is *still* that large is not asked at all.

### 6.3. General and specific signs for the presence of other or remaining systematic errors

Many specific signs of systematic errors for the individual data sets have already been discussed above. In this section an attempt is made to summarize the most important systematic errors. Signs of errors that are not specific to low-energy contamination are emphasized. These may interfere with the correction procedures. For an overview see Table 3 ▸.

(i) Clear signs of overfitting by too-large σ(*I*_o_) were observed in data sets **2_uncorr**, **2_corr**, **3_filter**, **4_uncorr**, **4_corr** and **4_filter**.

(ii) Signs of extinction (or detector saturation or shadowing) were found for reference data sets **5_uncorr**, **5_corr** and **5_filter** in the corresponding scatter pots of *I*_o_ versus *I*_c_.

(iii) Remaining outliers are found in all NPPs before and after correction, as well as for the filtered data sets.

(iv) The necessity of invoking a weighting scheme already implies a systematic error with the s.u. values or the model or both. A weighting scheme was applied in all data sets, in some cases with weighting scheme value *b* > 1 and up to *b* = 6.05 (for data set **3_uncorr**).

(v) Large agreement factors were found prior to and after 3λ correction, as well as for the filtered data sets, *e.g.* for reference data sets **1**, **2** and **4**. In data set **1**, the agreement factor is just lowered from 12.65% to a still quite high value of 11.13%. For the filtered data it remains at a high level of *wR*(*F* ^2^) = 11.05%, and similarly for data sets **2** and **4**. This may be a hint that other systematic errors are present in these sets. Data sets **1_uncorr**, **1_corr** and **1_filter** show the most significant 2λ contamination signals (with significances 1.83, 1.35 and 1.39, respectively), although these are all less significant than three standard deviations. This may point either to weak additional 2λ contamination or to other errors, which increase the 2λ contamination signal and may influence the correction procedure by adding a residual Δ_2λ_ to those reflections which are simultaneously multiples of two and of three. This leads to overcorrection, which is visible in a significantly increased contribution of *m*_3_ to the 10% (and 20%) most *negative* residuals in the corresponding histograms of data set **1_corr**. It is shown below that, for example, disorder can artificially increase a 2λ contamination signal.

Another cause of quite large weighted agreement factors is again too-large σ(*I*_o_) (Henn, 2019 ▸).^3^ Other causes could also still be at work. Overfitting by too-large σ(*I*_o_) was found with the help of the aGoF for all reference data sets **2** and **4** as mentioned above in point (i), but not for reference data set **1**. For data set **4**, as will be discussed below, there may additionally be a slight disorder. This can be detected with the help of the fractal dimension plot, where unmodelled disorder appears as a shoulder in the positive residual density [see *e.g.* Dittrich *et al.* (2018 ▸) and Meindl & Henn (2010 ▸)].

Each of these other unknown systematic errors may interfere with the correction procedure and lead to over­compensation, under­compensation or partial error compensation of other errors rather than low-energy contamination. In this case, the decrease in *wR*(*F* ^2^) after correction can be attributed only partially to the correction of low-energy contamination.

In order to evaluate the ‘costs’ of applying a weighting scheme in terms of the weighted agreement factor, one may compare the actual agreement factor with the s.u.(*I*_o_)-based predicted agreement factor (Henn & Schönleber, 2013 ▸; Henn & Meindl, 2014*a* ▸; Henn, 2019 ▸). The predicted agreement factor based on s.u.(*I*_o_) is the value that could be attained if there are no systematic errors at all, *i.e.* the s.u.(*I*_o_) are assumed to be adequate and GoF = 1.00. For data set **1_uncorr**, 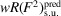 = 1.32%. This exemplifies the large costs for the application of a weighting scheme in reference data set **1**. The s.u.-based predicted agreement factors for reference data sets **2_uncorr**, **3_uncorr**, **4_uncorr** and **5_uncorr** are 1.11, 1.72, 2.73 and 1.33%, respectively. Comparing these values with the weighted agreement factors in Table 2 ▸, it is seen that there is a large gap between the potential of the data sets and their actual values in the agreement factors, similar to the *R*-factor gap in macromolecular crystallography (Holton *et al.*, 2014 ▸). Either it is not known how to determine the s.u.(*I*_o_) correctly, such that they nearly always need a correction *via* application of a weighting scheme, or the remaining errors in all these data sets are much larger than the error from low-energy contamination. Both cases are problem­atic.

(vi) The scatter plots of observed versus calculated intensities show unexpected and unexplained features. For example, in reference data set **4**, the strong intensities have a distinct tendency to be larger than the corresponding calculated intensities prior to and after the correction process, as well as for the filtered data set. A similar, but not as distinct, pattern is observed in reference data set **3**, which shows modelled disorder. It is not clear how this interferes with the correction procedure. However, it is clear that it *might* interfere with the correction procedure as (*a*) it clearly violates the assumption that the unaffected intensity can be replaced by a calculated intensity that is unbiased on the true intensity and (*b*) it adds to Δ such that this may again influence the value of *k*_3λ_, in particular when many *m*_3_ are affected by the error in a systematic way or when some of the *m*_3_ reflections are affected particularly strongly. The underlying cause for this error is also not clear. It may be connected to undetected or only partially modelled disorder.

A single large outlier for the strongest intensity is visible in **1_uncorr**, **1_corr** and **1_filter**.

(vii) There is possible slight disorder in data sets **3** and **4**. For data set **3** a disorder was modelled, but it may not be modelled completely. With weighting scheme parameters as large as *b* = 6.05, 4.60 and 3.62 for data sets **3_uncorr**, **3_corr** and **3_filter**, respectively, these remain very high.

(viii) All data sets show a distinct resolution-dependent error, which is indicated by the BayCoN plots 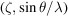 and 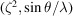 and the corresponding χ^2^ values. In each set the respective 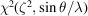 value is the largest from all χ^2^(ζ^2^, *X*), 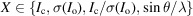 . This may indicate a common problem with the data acquisition or data processing steps. This observation is important, as it was found that, among the analysed χ^2^(ζ^2^, *Y*) values for the 127 data sets, those for 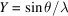 were the largest. The average values for the 127 data sets were 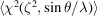 = 297.48, 〈χ^2^[ζ^2^, σ(*I*_o_)]〉 = 135.62 and 〈χ^2^(ζ^2^, *I*_c_)〉 = 221.35. There seems to be a widespread unknown resolution-dependent systematic error present in the overwhelming majority of these 127 analysed data sets. None of the data sets **1**–**5** shows a χ^2^ value smaller than 150 for the BayCoN plots 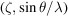 . For all members of reference data set **4** (**4_uncorr**, **4_corr** and **4_filtered**) these values are even above 1000. The corresponding values for all members of reference data set **5** are between 451.90 (**5_uncorr**) and 455.40 (**5_corr**), which are also much higher than the threshold value of 149 (Henn & Meindl, 2014*b* ▸). This disproves the uniformity of the corresponding plots and establishes a systematic (nonlinear) connection between the residuals (and squared residuals) and the resolution.

(ix) All members of reference data sets **4** (**4_uncorr**, **4_corr** and **4_filter**) and **5** (**5_uncorr**, **5_corr**, **5_filter**) show much larger χ^2^ values for the (ζ, *X*) standard BayCoN plots compared with the (ζ^2^, *X*) standard plots, 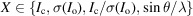 . From the large χ^2^ values for the (ζ, *X*) plots, those for 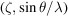 are by far the largest. This might suggest that a primary resolution-dependent systematic error induces as a secondary effect the non-uniformity of the remaining BayCoN (ζ^2^, *Y*), *Y* ∈ {*I*_c_, σ(*I*_o_), *I*_c_/σ(*I*_o_)}, plots.

Data set **4** is of particular interest as it shows, simultaneously, overfitting by too-large σ(*I*_o_) *and* large χ^2^(ζ, *X*) values. Too-large σ(*I*_o_) lead to artificially uniform BayCoN plots. Nevertheless, the χ^2^(ζ, *X*) values are still all much larger than 149. Data set **4** also has by far the most reflections. The χ^2^ statistics are more sensitive the larger the number of reflections. As it has already been pointed out that all data sets show a resolution-dependent error, it could be the case that this error just becomes particularly visible due to the large number of reflections in data set **4**. The resolution-dependent error seems to be of great importance as it appears in all data sets. It is therefore described briefly in the next section.

### 6.4. Brief characterization of the resolution dependence

**Figure 5:**
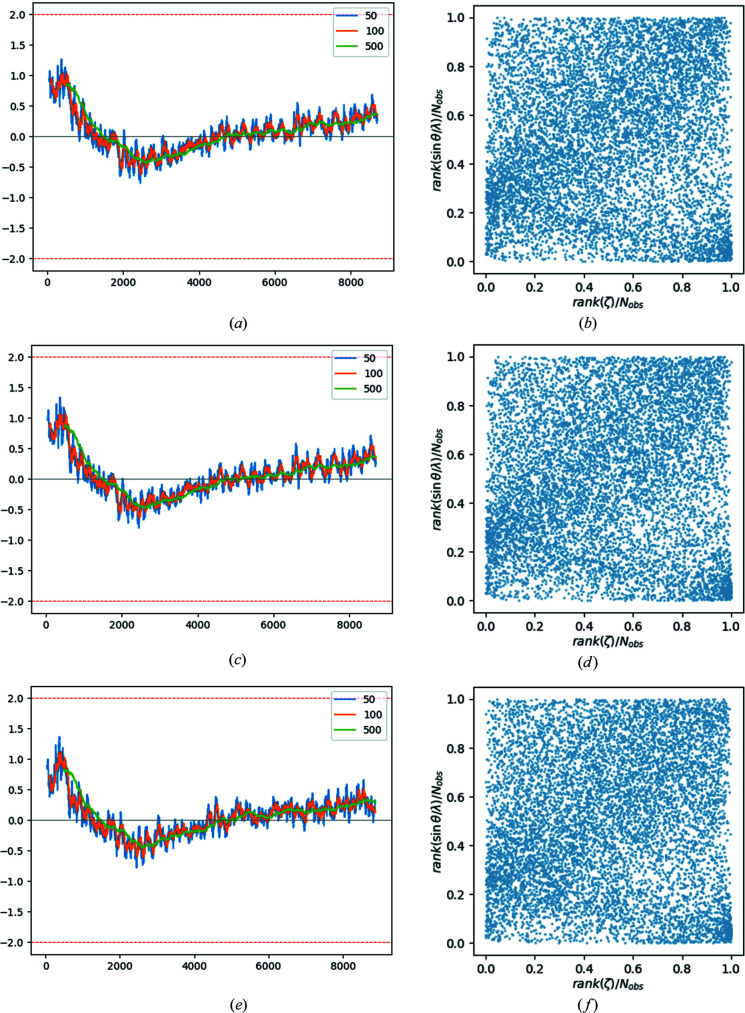
Data set **4**. The left-hand column shows moving averages of the residuals sorted in ascending order of resolution, and the right-hand column shows the corresponding BayCoN plots 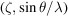 . The moving averages are calculated for windows of 50 (blue), 100 (orange) and 500 (green) consecutive reflections for (*a*) the uncorrected data set, (*c*) the corrected data set and (*e*) the filtered data set. They all show the characteristic ‘spoon’ form of initially quickly decreasing and then slowly increasing mean values, which was also found frequently in the 127 data sets analysed earlier. The corresponding BayCoN plots are also virtually unaffected by the correction procedures. The 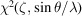 values are (*b*) 1208.02, (*d*) 1265.37 and (*f*) 1023.96, all of which are far larger than the threshold value of 149.

In order to describe the resolution dependence of the residuals, two types of plots are chosen (depicted in Fig. 5 ▸) and briefly discussed for the example reference data set **4**. The first type of plot shows the moving averages of the residuals for different averaging windows, sorted by resolution. In all three cases (**4_uncorr**, **4_corr** and **4_filter**) the same pattern appears: in the beginning there is very steep decrease from a positive region to a negative region, followed by a steady and slow increase again. The overall form is reminiscent of a spoon. This spoon-like pattern, or a similar pattern starting from the negative region with a steep increase to positive values and a slow decrease again to negative values (inverse spoon), was observed frequently in 127 data sets from *IUCrData* (not shown). It seems to point to a common error. The corresponding BayCoN plot 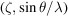 also shows a typical pattern, in which the weighted residuals are highly non-uniformly distributed. For reference data set **4_uncorr**, the residuals are strongly polarized towards large positive values for the lowest-resolution shell, as indicated by the high concentration of points in the lower right-hand corner of the plot [Fig. 5 ▸(*b*)]. For slightly larger resolution values, the polarization of the residuals reverses to negative values and from there the highest density of points moves slowly again to the top right-hand side for increasing resolution. This pattern is virtually the same for all members of data set **4**, *i.e.* it is in essence not affected by the correction procedure or filtering. Reference data sets **2**, **3** and **5** show similar patterns, and reference data set **1** shows the pattern of the inverse spoon.

This common phenomenon could be caused by a slight nonlinearity in the detector response to a large dynamic range, but this is speculative at this point and deserves to be investigated in greater detail. The widespread resolution dependence might explain why empirical correction methods based on resolution-dependent scale factors seem to be able to reduce the agreement factors (Niepötter *et al.*, 2015 ▸), although it would be better to learn how this error could be avoided in the first place.

## 7. Other causes of low-energy contamination signals

In order to have a unique signal of low-energy contamination, it must be shown that the metrics used to detect low-energy contamination do not lead to ‘false positive’ results, or, if false positive results exist, the circumstances for false positive results need to be characterized. The question is whether there are circumstances that lead to a significant increase in *m*_*x*_ (*x* ∈ 2, 3, 6) to rare events |ζ| > 3 *without* low-energy contamination.

In the book *Crystal Structure Refinement: A Crystallographer’s Guide to SHELXL* edited by Peter Müller (2006 ▸), examples for disordered and twinned structures are given and discussed in detail. The *SHELX* input files are given for each individual step in modelling of disorder and twinning, starting from scratch, where the problem is usually not yet known, and leading to the final models in which disorder and twinning are modelled. This provides a great opportunity to use examples known by a broad audience and processed by renowned specialists.

### 7.1. Twinning

From these examples, in the first of two discussed cases of twinning by reticular merohedry, a distinct signal for 3λ contamination appears. The detailed numbers are as follows: 93 reflections were multiples of three, *m*_3_, corresponding to 10.95% of all reflections. The *m*_3_ contributed seven rare events |ζ| > 3 from a total of 14, *i.e.* 50%. The deviation of the given fraction from the expected fraction corresponds to a 2σ event. A polarization of the residuals with respect to *m*_3_ is also visible in the corresponding histogram. After modelling twinning in data set **ret1-03**, the 3λ signal is insignificant: the *m*_3_ contribute only two rare events |ζ| > 3 from a total of 12 rare events, *i.e.* 16.67%. This is less than one standard deviation from the expected 10.95%.

Twinning by reticular merohedry may therefore lead to an increase in the significance of 3λ signals that decreases again after modelling of twinning. In the second example for twinning by reticular merohedry, *m*_2_, *m*_3_ and *m*_6_ all contribute to the rare events as expected, prior to and after modelling of twinning.

### 7.2. Disorder

In the section about disorder, the example of a Ti^III^ compound is discussed. The initial model **Ti-01** leads to a 2λ signal with significance 7.65, *i.e.* high significance. Modelling of the disorder in **Ti-07** reduces the significance of the 2λ signal to 0.34, *i.e.* it is insignificant. In greater detail, the numbers are as follows. In **Ti-01**, 1156 reflections (27.03%) are multiples of two, *m*_2_. These contribute 655 rare events |ζ| > 3 to a total of 1699 rare events (38.55%). The difference is 11.52 percentage points, which is equivalent to a 7.65σ event.

Disorder in the discussed example resulted in a significant 2λ signal which vanished after modelling of disorder.

A particularly interesting example of disorder is given by the solvent molecule toluene, where the second position is twisted by about 180°. File **tol-01** shows a 2λ signal with significance 2.01 that becomes *more* instead of less significant after modelling of disorder (significance 3.44) in **tol-05**. Among the examples of disorder discussed here (Ga, Ti, toluene and benzene), the disordered toluene structure is the only one that also shows a fractal dimension plot with large shoulders after modelling of disorder. This points to another, undetected, systematic error in this data set, such as yet another disorder. In all other cases of disorder discussed, the fractal dimension plot is parabolic in shape after modelling of disorder.

### 7.3. Inadequate standard deviations of the observed intensities

Finally, inadequate standard deviations of the observed intensities may also lead to false positive and false negative low-energy contamination signals. This can be deduced easily by just thinking about what happens when all standard deviations are too small or too large by the same factor. When they are all massively too large, this will eventually lead to zero rare events, such that the multiples of two, three or six are also not able to contribute to rare events. As a possible example for this, the case of pseudo-merohedic twinning in data set **pmero-02** shows only one rare event from multiples of two and none at all from multiples of three or six. The percentage of multiples of two with 273 reflections is 15.25%, but all *m*_2_ contribute only one rare event corresponding to 1.92% of all rare events. So 15.25% of reflections contribute 1.92% of rare events. There are further hints of too-large σ(*I*_o_) in this data set, such as 〈|ζ_−_|〉 = 0.65 ± 0.06 (3σ) being significantly too small compared with the reference value [(2/π)α]^1/2^ = 0.73, and similarly for 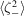 = 0.74 ± 0.17, which is also significantly smaller then its reference value α = 0.92.

For *too-small* average values one reason could be too-large standard deviations. Another reason could be that the mean value of the residuals is significantly shifted to positive values, which is also the case in this data set: the shift of the mean of residuals at 〈ζ〉 = 7.56σ(〈ζ〉) is highly significant, as is the excess number of positive residuals [(#ζ_+_ − #ζ_−_)/(*N*_obs_)^1/2^ = 3.55]. It would require a much more detailed analysis to (dis)prove inadequate standard deviations as the cause for a substantially reduced signal of low-energy contamination that may lead to false negative results, but this is out of the scope of this work. Nevertheless, the above *Gedankenexperiment* of having too-large standard deviations proves the relevance of inadequate standard deviations.

The other extreme is when the σ(*I*_o_) are much too small, as this will lead to an abundance of rare events and in consequence to small error bars as derived from Poisson statistics. This would make even small deviations from the expected value suddenly significant, although that significance would be artificial. A fingerprint trace of this error could be that all signals *m*_2_, *m*_3_ and *m*_6_ are simultaneously (significantly) large. There was no example of this case in the discussed data sets.

The whole question of how flawed σ(*I*_o_) influence low-energy detection (and the detection of other systematic errors) is a big topic that definitely needs more attention.

For now, it can be accepted that it is plausible that at least grossly flawed σ(*I*_o_) may influence the detection of low-energy contamination adversely by leading to false positive and false negative results.

## 8. Other causes for significant positive shifts of the mean value of the residuals

As it is expected that low-energy contamination will be accompanied by an increased frequency of positive residuals and stronger positive residuals and by a shift of the mean value of the residuals to positive values, and as disorder, twinning and flawed standard deviations may lead to false positive low-energy contamination signals as given by significantly increased percentages of *m*_2_ and *m*_3_ to rare events |ζ| > 3, it is worth asking how disorder and twinning affect the mean value of the weighted residuals.

### 8.1. Twinning

Modelling of twinning shifts the mean value of the residuals to lower values in all cases where initially a significant positive shift was given. For the merohedric case, the shift is quite substantial, from a highly significant 19.84 to an insignificant −0.47, and similarly in the pseudo-merohedric case, where the shift is from a highly significant 7.56 to an again insignificant −0.78. In other cases, a substantial downward shift results in a still significant value like for reticular case **ret2**, where the initial very significant value (20.22) is still significant after modelling of twinning (3.69). In the nonmerohedric case **nmero2** all the values are insignificant. Of particular interest is the case of nonmerohedric twinning **nmero1**, where the shift is from a significant 5.98 to an again significant but negative −4.92. A similar, but not as distinct, tendency is found for the significance of positive excess residuals, which are reduced for the merohedric case from 9.88 to 2.64, for the pseudo-merohedric case **pmero** from a significant 3.55 to an insignificant −0.24, and for reticular twinning **ret2** from a highly significant 11.04 to a still significant 4.33. In the nonmerohedric case **nmero2** all values are again insignificant, whereas in **nmero1** the value for unmodelled twinning of −5.46 is significantly negative (significant excess number of negative residuals) and remains significantly negative at −5.67 after modelling of twinning (Table 4 ▸).

### 8.2. Disorder

Table 5 ▸ gives the deviation of the mean value of the residuals from zero for data sets with unmodelled and modelled disorder, as well as the significance of the number of positive excess residuals for unmodelled and modelled disorder. 〈ζ〉/σ(〈ζ〉) is shifted to significant positive values in all cases where disorder is not modelled, and it is reduced in all cases except toluene when disorder is taken into account in the model. In the toluene data set there are still other significant systematic errors present, as can be seen from the broad residual density distribution (see the fractal dimension plots in the supporting information). It is concluded that it is very likely that un­modelled disorder easily leads to a positive shift in the mean value of the residuals, in particular when it is not disorder about special positions.

## 9. Validation of the results with other data

Macchi *et al.* (2011 ▸) discussed the problem of low-energy contamination in the context of sealed tubes with multilayer optics. Contaminated data sets were chosen and model refinements were compared with data sets with a thin aluminium filter to block low-energy radiation.

**Figure 6:**
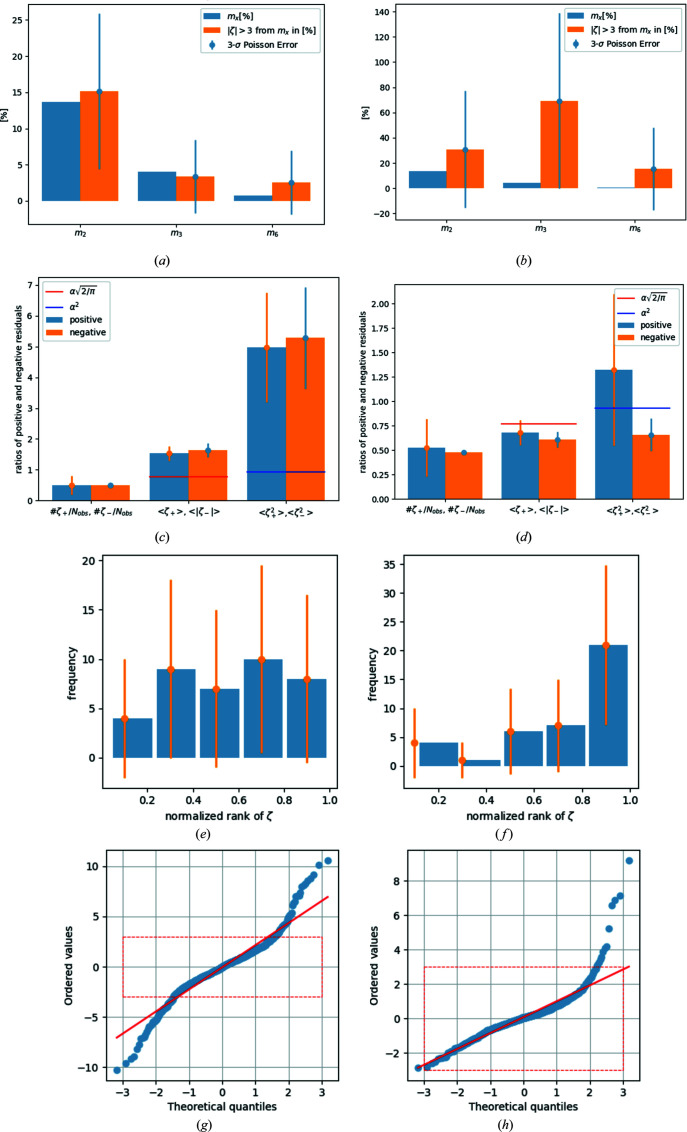
Data sets **IA** (filter, left-hand column) and **IB** (no filter, right-hand column) from Macchi *et al.* (2011 ▸). All signs of 3λ contamination vanish for the filtered data set **IA**, as expected: (*a*) the 3λ signal is insignificant, (*c*) the positive squared residuals are no longer on average much larger than the negative ones, (*e*) the histogram of the number of *m*_3_ in different bins of the residuals is uniform and (*g*) the NPP still shows outliers, although it is not one-sided any more. Panels (*b*), (*d*), (*f*) and (*h*) show the corresponding plots for **IB**. In panel (*b*) the initial 3λ signal is just not significant despite proven 3λ contamination in this set. Too-large σ(*I*_o_) from a large weighting scheme parameter *a* = 0.10 prevent the signal from becoming significant (for more information see text).

Fig. 6 ▸ shows a number of plots for a data set contaminated with low-energy radiation (data set **IB**) and the corresponding filtered data set **IA**. The percentage contributions of *m*_2_, *m*_3_ and *m*_6_ to the rare events are all increased compared with the expected contribution [Fig. 6 ▸(*b*)], but none is significant according to a 3σ criterion (the significance of the *m*_2_ signal is 1.11, that of *m*_3_ is 2.82 and that of *m*_6_ is 1.35). But this data set was chosen because it is known to be contaminated from observation of typical radial streaks in the reconstructed diffraction images. The one-sided NPP [Fig. 6 ▸(*h*)] and 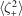 = 1.32 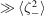 = 0.66 [compare with Fig. 6 ▸(*d*), right] indicate low-energy contamination. The histogram of the number of *m*_3_ in five different bins of weighted residuals ζ shows a peak for large positive residuals, which again confirms 3λ contamination [Fig. 6 ▸(*f*)].

The balance sheet in Fig. 6 ▸(*d*) (middle) shows that the mean values for positive and absolute negative residuals are both below the expected value, which is interpreted as a sign of σ(*I*_o_) being generally too large in this data set. This finding may not be surprising in view of the large weighting scheme parameter *a* = 0.10, resulting in unweighted squared residuals being on average less than half the mean squared standard deviation, 〈Δ^2^〉 = 0.47〈σ^2^〉, and a correspondingly low aGoF = 0.70, *i.e.* overfitting. However, GoF = 1.02 fails to indicate overfitting. (Note that the weighting scheme is constructed with the purpose of bringing the GoF close to one, so it does a good job for that specific purpose, but this is in conflict with the purpose of GoF to indicate systematic errors.)

The (too-)large standard deviations lead to a correspondingly small number of rare events in this data set: in total there are 13 rare events |ζ| > 3, four from *m*_2_, nine from *m*_3_ and two from *m*_6_. Due to the small number of events, the corresponding Poisson-based standard deviations are large, which leads to an insignificant 3λ signal, despite the large difference between the expected (4.17%) and observed (69.23%) contributions of *m*_3_ to |ζ| > 3. This data set is an example for the further above-mentioned situation in which too-large standard deviations suppress the significance of the low-energy contamination signal. The corresponding filtered data set **IA** does not show, as expected, any of the discussed signs of 3λ contamination.

## 10. Relevance of low-energy contamination and yet another means of detection

When searching for data sets with low-energy contamination, one faces mainly two problems with the metrics: (i) The standard uncertainties are questionable, in particular when large weighting scheme parameters are employed. Too-large standard uncertainties may artificially lead to an insignificant contribution of *e.g.* *m*_3_ to all rare events. (ii) The chosen criterion of using a 3σ Poisson-based error bar as a threshold value for detection of low-energy contamination may be too rigid. Not even all reference data sets known to be contaminated by low-energy radiation exceeded this threshold value: reference data set **5** showed a significance of only 2.14 for 3λ contamination, as can be seen from Table 2 ▸. Another problem is with the interpretation of the metrics, as 3λ-contamination signals may also indicate other errors like twinning or disorder as discussed above. One way of solving these problems with the metrics is again to use the histograms of contributions from *m*_3_ to the residuals, as used and discussed above.

Another way of quantifying this could be to look additionally at the ratio of expected and observed contributions of *m*_3_ to rare events. If the observed ratio is distinctly larger than one, *e.g.* larger than two, but insignificant by the 3σ criterion, it might be a good idea to investigate more deeply into this data set. One way of deepening the investigation is to ask whether there are hints of overestimated σ(*I*_o_), which may artificially make a 3λ contamination signal insignificant.

As an example, in a pyrazoline study by Yoo & Koh (2021*a* ▸), the ratio of observed to expected contributions of *m*_3_ to rare events is 8.37, while the significance based on Poisson statistics is, with a value of 2.49, lower than 3σ. There are multiple signs of overestimated σ(*I*_o_) in this data set, like aGoF = 0.77 < 1.0, which are not further discussed here. This may be an example of a data set with undetected 3λ contamination due to too-large σ(*I*_o_) values. A similar situation arises with the data set used by Ovalle *et al.* (2021 ▸), with a ratio of observed versus expected contributions of *m*_3_ to rare events of 2.07, *i.e.* the multiples of three contribute to the rare events twice as often as expected. With only five rare events from multiples of three, this signal remains insignificant by the 3σ criterion. There are again, however, hints of overestimated σ(*I*_o_), as given *e.g.* by aGoF = 0.62. Both mentioned data sets show one-sided NPPs and further signs of low-energy contamination.

Going through all 94 data sets published by *IUCrData* in 2021, 23 data sets (24%) show a ratio of observed to expected contributions of *m*_3_ to rare events larger than two. A list of the affected data sets with the corresponding numbers and plots is given in the supporting information. Among these data sets are two that used Cu radiation, for which a 3λ contamination is not expected, as this is supposed to happen only with Mo radiation in combination with mirror optics. Only one of the 94 data sets shows a significance of contributions of *m*_3_ to rare events larger than three (Yoo & Koh, 2021*b* ▸), a study of an isoflavone. This is also the data set that shows the largest ratio of 12.52 of observed to expected contributions of *m*_3_ to rare events. This data set may be re-examined by the authors and tested for low-energy contamination and other systematic errors like disorder, twinning or phase transitions, which may also lead to a low-energy contamination signal. Other data sets that may profit from re-examination with respect to low-energy contamination signals are a study of a cyclo­hexyl­idene derivative from Sivapriya *et al.* (2021 ▸) (ratio of observed and expected contributions of *m*_3_ to rare events 6.45, significance based on Poisson statistics 2.67), a re-determination of barium bis[tetra­fluorido­bromate(III)] from single-crystal diffraction (measured with Cu radiation, which makes it unlikely to be caused by 3λ contamination, as this is expected only for Mo radiation in combination with mirror optics) instead of a powder diffraction experiment by Ivlev & Kraus (2021 ▸) (ratio 6.05, significance 2.89), and a study of a tri­phenyl­amine derivative by Patel *et al.* (2021 ▸) (ratio 5.91, significance 2.88). These findings again seem to suggest that a 3σ Poisson statistics based criterion for the detection of low-energy contamination signals may be too rigid as serious signs of low-energy contamination start appearing earlier, in this subset of 95 data sets at approximately 2.5σ.

The examples discussed again stress the importance of the correctness of the σ(*I*_o_). As long as this problem is not solved and weighting scheme parameters are used to disguise errors, real and substantial progress in increasing overall data quality and the accuracy of the models is prevented. Already the detection of systematic errors is hampered.

## 11. Open questions

The present work leads to general and specific questions that need to be answered by the crystallographic community. The specific questions are connected to low-energy contamination:

(i) What is the correct procedure when signals for 2λ and 3λ contamination are simultaneously present?

(ii) What is the correct procedure when extinction is present? Does the extinction correction need to be applied first and only then low-energy contamination correction, or the other way round?

(iii) What is the correct procedure when overfitting by too-large σ(*I*_o_) is present? The general problem here is that it may not be obvious from the start that overfitting is present, as overfitting by too-large σ(*I*_o_) may be counteracted by other systematic errors such that GoF and aGoF result in values very close to one despite the presence of other errors.^4^ In the present case overfitting was obvious from aGoF < 1 for the affected data sets. But as systematic errors tend to increase the unweighted residuals, such that 〈Δ^2^〉 increases, this may result in aGoF > 1 and overfitting may still be present, but obstructed by other systematic errors. Overfitting can be detected in these cases only after removal of one or more systematic errors, all of which increase 〈Δ^2^〉. Another problem is that empirical correction procedures employing too-large σ(*I*_o_) may lead to undercompensation. This point touches on the more general topic that there is still no commonly accepted procedure for testing the σ(*I*_o_), which would also be important for the validity of the least-squares procedure.

The more general questions are:

(i) How does one go about correction procedures in general? There is no common guideline for this. It is just tacitly assumed that, if an error occurs like a low-energy contamination, it can be corrected for. But the present work raises some doubts: systematic errors may interact and lead to over- or under­compensation, as well as to incomplete correction of other errors rather than the intended one. In practice this means that some correction procedures may introduce new and more systematic errors than they can possibly remove. It is not yet common practice to investigate the necessary requirements for a valid and helpful correction procedure. The authors’ personal view is that this lack is due to insufficient instruments for error analysis and error description in diffraction data, but it is still a good idea to monitor the circumstances and requirements to be able to discriminate between cases in which the systematic error is decreased by a correction procedure and cases in which it is not. Monitoring only *wR*(*F* ^2^) and the GoF is not sufficient for this. They may both be affected by flawed σ(*I*_o_). Monitoring errors in the bonding distances would be helpful if the total error were calculated and not just the statistical error. The total error is composed of a systematic error and a statistical error, but to this day, the systematic error in the model parameter values is rarely evaluated. Due to high redundancies, the systematic error may be the dominant error nowadays, even in small-molecule crystallography. It is clear that *e.g.* 2λ contamination signals, extinction, detector saturation, or too-small or too-large σ(*I*_o_) all hamper or even prevent the correct functioning of a 3λ correction procedure, yet in practical applications this is often not monitored.

(ii) When the correction of a systematic error reduces *wR*(*F* ^2^) from 12.65 to 11.13%, like in reference data set **1**, but the expected agreement factor for absence of all systematic errors (including those that lead to non-zero weighting scheme parameters) *wR*(*F* ^2^)^pred^ = 1.32% is one order of magnitude smaller, should this not ring alarm bells? Is it really helpful to perform a small correction reducing *wR*(*F* ^2^) by 1.52 percentage points and disregard the causes of and potential interactions with the remaining errors that could reduce the weighted agreement factor by 11.33 percentage points?

(iii) Why are weighting scheme parameters like *b* > 1 commonly accepted without even discussing the possible causes? Either the s.u. values are grossly wrong or there is a problem with the model; in either case, action is required. The first important step could be to identify the error as one in the model or in the s.u. values. But none of these problems are addressed. This is again a common problem, as was pointed out earlier [see, for example, the discussion on pages 140 and 141 in the article by Henn (2019 ▸)].

For low-energy contamination it seems to be important always to monitor 2λ and 3λ contamination together prior to and after the correction procedure. Histograms of multiples in bins of residuals may be helpful for diagnostic purposes and can reveal overcompensation processes like in reference data set **1**, or processes in which 2λ contamination becomes more significant only after 3λ correction like in reference data set **2**.

## 12. Summary and concluding remarks

A number of new data quality descriptors have been introduced to tackle the problem of low-energy contamination. Among these are the significance of the deviation of the residuals from zero, the separate mean values of positive and negative residuals with their reference values and error bars, the separate mean values of the squared positive and negative residual values with their reference values and error bars, and the significance of the low-energy contamination signals based on Poisson statistics, together with histograms of the multiples in bins of the weighted residuals and squared weighted residuals.

*A priori* expectations about traces of low-energy contamination in the fitted data have been formulated and compared with the experimental findings. Some of the expected features were found consistently in the affected data sets, some not. In this case it is assumed that the expected features were obstructed by other systematic errors. This detailed comparison facilitated discrimination between robust and ‘fragile’ signs of low-energy contamination, which are easily obstructed or even reversed by other systematic errors. The concept of primary and secondary effects was applied to the question of whether low values in 〈|ζ_−_|〉 and 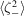 are a sign of too-large standard deviations (primary cause) or an effect of the shift of the mean values of the residuals to a positive value (secondary cause). The origin of the positive shift of the mean value of the residuals in all data sets **1**–**5** remains unclear. It is most likely that many different systematic errors (disorder could be one of these) lead to positive shifts in the residuals. It is important to investigate this, as many data sets show a significant positive shift of the mean value of the weighted residuals.

In the view of the present authors, a detailed list of expected effects for a given systematic error on positive and negative weighted residuals, as done here for low-energy contamination, should be compiled for every relevant systematic error. This is invaluable for discriminating between robust and fragile signs and for learning how systematic errors are connected and how they affect each other. This diagnostic procedure will be very helpful in identifying and removing systematic errors at a later stage, but diagnosis is prior to the cure and the art of diagnosing systematic errors seems not to be well developed yet in crystallography when, as an example, weighting scheme parameters like *b* > 6 are left unmentioned, undiscussed and undisputed. The present work aims to contribute to the development of diagnostic standards and protocols. For all metrics used to describe the fit quality and systematic errors, it should be established under which circumstances they are applicable [to start with the simplest: are *wR*(*F* ^2^) and the GoF applicable in cases of flawed standard deviations?] and when they are not, and how to monitor these circumstances. This question is related to the question of false positive and false negative results, which should be discussed for all metrics as well (as an example: too-large standard deviations may lead to a low GoF value, despite the presence of systematic errors), and to error compensation processes. It would also be helpful to discuss the nature of the appearance of systematic errors in terms of whether they are primary or secondary. If they are secondary, the primary error may still be unknown and a search for suitable candidates may follow. The reduced mean values of negative residuals are an example here: a primary error could be that the standard deviations are too large, leading to overall reduced residuals, which are then increased again by the systematic error of low-energy contamination only for the positive residuals. A secondary effect would be that the negative residuals are just reduced, due to a residual distribution that is, on average, shifted to positive values as a whole, leading to a large positive significance of the mean value of the residuals, which was found in some of the data sets as well. We have mentioned that primary errors leading to positive shifts in the residuals are low-energy contamination, disorder and twinning, but there may be many more.

The present work needs to be seen within a much wider framework than just low-energy contamination, as it touches on some important questions which are relevant for all correction procedures, including those at the data processing level and for the treatment of systematic errors in general. The authors’ personal view is that it is important to discuss the appearance of systematic errors in as much detail as has been done here, by breaking it down into such simple questions as how a specific systematic error affects the NPP, the separate positive and negative mean values of the residuals, the mean values of positive and negative squared residuals, the significance of the deviation of the mean value of the residuals from zero and so on. Analysing systematic errors in such great detail leads to a steep increase in knowledge of systematic errors, and in the long run will help to improve experiments in terms of cost, precision and accuracy. Therefore, what this needs is to put systematic errors, and their metrics, appearance and interactions, at the centre of attention. This will help to clarify under which circumstances large weighting scheme factors appear, in which cases these are due to flawed s.u.(*I*_o_) and in which cases the model is flawed, what kind of errors lead to *I*_o_ > *I*_c_ in the corresponding scatter plots, and why there is an all-pervasive systematic error with respect to the resolution in many published data sets, to name just a few. Many insights gained in this context may help to improve not only standard experiments but also high-resolution and macromolecular experiments, and may be at least partially transferable to neutron and even to electron diffraction experiments.

Other questions are: How do systematic errors interfere with each other? Which other systematic errors need to be *excluded* in order to have a reasonable empirical correction procedure that does not account for those other errors? When do correction procedures reduce the total number of systematic errors and under which circumstances are these unintentionally increased, despite *e.g.* lower agreement factors and lower GoF values? What are the adequate metrics to quantify the total number of systematic errors in a given data set? These are also needed to quantify progress. Metrics like GoF and *wR*(*F* ^2^) are problematic as they fail when too-large (or too-small) σ(*I*_o_) are involved. Their failure to serve as objective metrics may not be obvious. A lower value of both metrics after application of a correction procedure may be attributed to partial correction of other remaining errors. This leads to the question: When is a reduction in the agreement factor or in the GoF correctly solely attributed to a correction procedure, and under which circumstances is this invalid? This question goes rather deep and cannot be answered fully here. It is obvious that some sort of assessment of the remaining systematic errors and their interaction with the correction procedure is needed, but this is rarely done. It would also be helpful to know more about the hierarchy of errors, *i.e.* about the level at which the errors appear (data acquisition, data processing, model refinement). From the appearance of a similar resolution-dependent error in all reference data sets studied here (and in many others from other authors), it seems to be a plausible hypothesis that this error appears at a more fundamental stage such as the data acquisition or data processing steps. How can this be verified or falsified?

Our closing questions are: Can the indicators for low-energy contamination be improved further? When will crystallographic diffraction experiments finally be equipped with reliable standard uncertainties for the observed intensities? When will crystallographers at least start to discriminate between the case where systematic errors are in the s.u. values, which makes some form of correction procedure necessary like the application of a weighting scheme, and the case where the s.u. values are adequate and the resulting differences are due to other model errors. As the s.u. values are entering the data quality evaluation process, for example in the weighted agreement factor and the GoF, this distinction would be essential for progress in the field of data quality assessment and improvement.

## 13. Related literature

The authors of additional data sets cited in the supporting information are as follows: Abou *et al.* (2021 ▸); Castaldi *et al.* (2021 ▸); El-Hiti *et al.* (2021 ▸); Ha (2021*a* ▸,*b* ▸,*c* ▸,*d* ▸,*e* ▸); Hu *et al.* (2021 ▸); Meenatchi *et al.* (2021 ▸); Pacifico & Stoeckli-Evans (2021 ▸); Sathya *et al.* (2021 ▸); Su *et al.* (2021 ▸); Sung (2021 ▸); Vinotha *et al.* (2021 ▸); Yaffa *et al.* (2021 ▸); Yang & Long (2021 ▸).

## Supplementary Material

Additional diagnostic plots and numbers for the remaining data sets. DOI: 10.1107/S1600576723004764/oc5025sup1.pdf

## Figures and Tables

**Table 1:** Details of data sets **1** to **5** discussed in this work

No.	Formula	X-ray source	*T* (K)	Absorption coefficient μ (mm^−1^)
**1**	C_28_H_18_N_2_	IµS	100	0.081
**2**	C_12_H_4_N_4_	IµS	100	0.088
**3**	C_18_H_17_CuO_6_	IµS	100	1.318
**4**	C_34_H_26_MgN_4_O_4_	TXS	100	0.111
**5**	C_11_H_10_O_2_S	IµS	293	0.294

**Table 2:** Characteristic numbers for low-energy contamination The first column gives the name of the data set, the second the percentage of multiples of three and the third the percentage of rare events from multiples of three (which should be close to the value in column two when low-energy contamination is not present). The absolute numbers are also given. Column four gives the absolute numbers of *m*_3_ in the class of the strongest 20% of residuals and the corresponding percentage, column five displays the significance of the shift of the mean weighted residuals from zero, and column six the significance of the positive excess residuals according to a random walk criterion with equal probability for positive and negative steps. Column seven shows a theoretical reference value from a Gaussian distribution for the mean values in the next two columns, which are the separate mean value of the positive weighted residuals (column eight) and the absolute mean value of the negative weighted residuals (column nine). These values in column eight and nine are equal within the limits of statistical fluctuations, and equal to the reference value in column seven when no systematic errors apply. Column ten shows a theoretical reference value from a Gaussian distribution for the next two columns, which display the separate mean values of the positive squared weighted residuals and of the negative squared weighted residuals. These two values are equal within statistical fluctuations and in accordance with the reference value from column ten when no systematic errors apply. Squaring the residuals emphasizes outliers. Column 13 shows the weighted agreement factor as a percentage value, column 14 gives the goodness of fit and column 15 the alternative goodness of fit. In columns eight, nine, 11 and 12, statistical fluctuations are indicated by a 3σ error bar.

Data set	*m*_3_ (%)	|ζ| > 3 from *m*_3_ (%) signif†	*#m*_3_ in largest 20% of ζ^2^	〈ζ〉/σ(〈ζ〉)	(#ζ_+_ − #ζ_−_)/(*N*_obs_)^1/2^	(2/π)^1/2^α	〈ζ_+_〉	〈|ζ_−_|〉	α^2^	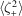	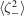	*wR*(*F* ^2^) (%)	GoF	aGoF
**1_uncorr**	3.54	50.00 (13/26) 3.35	28/60 (46.67)	3.85	1.00	0.73	0.81± 0.10	0.65± 0.06	0.85	1.57± 0.63	0.70± 0.04	12.65	1.12	1.05
**1_corr**	3.54	0.00 (0/18) –	15/60 (25.00)	2.96	1.29	0.73	0.81± 0.08	0.71± 0.06	0.85	1.34± 0.40	0.84± 0.05	11.13	1.09	1.14
**1_filter**	3.63	8.70 (2/23) 0.82	17/62 (27.42)	2.74	1.23	0.73	0.83± 0.08	0.74± 0.06	0.85	1.28± 0.27	0.93± 0.06	11.05	1.10	1.23
**2_uncorr**	3.69	41.38 (12/29) 3.15	24/57 (42.11)	2.33	1.40	0.76	0.76± 0.09	0.68± 0.07	0.91	1.35± 0.58	0.90± 0.07	10.75	1.09	0.90
**2_corr**	3.69	0.00 (0/27) –	16/57 (28.07)	1.44	1.76	0.76	0.78± 0.07	0.77± 0.08	0.91	1.10± 0.25	1.15± 0.09	9.63	1.09	0.98
**2_filter**	3.75	0.00 (0/26) –	11/58 (18.97)	1.10	1.70	0.76	0.77± 0.07	0.78± 0.08	0.91	1.06± 0.24	1.19± 0.10	9.92	1.08	1.00
**3_uncorr**	3.78	38.33 (23/60) 4.32	66/162 (40.74)	5.47	2.69	0.74	0.78± 0.05	0.67± 0.04	0.86	1.32± 0.32	0.78± 0.03	7.04	1.07	1.11
**3_corr**	3.78	12.24 (6/49) 1.69	44/162 (27.16)	4.03	2.78	0.74	0.77± 0.04	0.71± 0.04	0.86	1.08± 0.16	0.90± 0.04	6.50	1.03	1.09
**3_filter**	3.73	6.82 (3/44) 0.78	34/158 (21.52)	2.80	3.06	0.74	0.76± 0.04	0.75± 0.04	0.86	1.02± 0.15	0.96± 0.04	6.81	1.03	0.94
**4_uncorr**	3.61	27.54 (19/69) 3.79	90/314 (28.66)	6.40	4.10	0.76	0.80± 0.03	0.72± 0.03	0.91	1.17± 0.15	0.84± 0.02	11.48	1.03	0.79
**4_corr**	3.61	4.62 (3/65) 0.38	57/314 (18.15)	5.37	3.80	0.76	0.80± 0.03	0.75± 0.03	0.91	1.08± 0.10	0.88± 0.02	10.91	1.02	0.86
**4_filter**	3.67	2.82 (2/71) 0.43	66/325 (20.31)	5.74	3.44	0.76	0.81± 0.03	0.75± 0.03	0.91	1.14± 0.10	0.90± 0.02	11.13	1.03	0.86
**5_uncorr**	3.71	19.44 (7/36) 2.14	36/91 (39.56)	2.92	2.18	0.76	0.83± 0.06	0.77± 0.06	0.90	1.24± 0.22	0.99± 0.05	6.80	1.09	1.50
**5_corr**	3.71	3.33 (1/30) 0.11	24/91 (26.37)	2.36	2.50	0.76	0.81± 0.06	0.79± 0.06	0.90	1.17± 0.20	1.03± 0.05	6.68	1.08	1.47
**5_filter**	3.71	3.57 (1/28) 0.04	19/91 (20.88)	2.58	1.27	0.76	0.85± 0.06	0.78± 0.06	0.90	1.27± 0.22	1.03± 0.05	6.74	1.10	1.46

† The significance of the 3λ signal is calculated by Δ_%_/σ_Poisson,%_, where Δ_%_ is the difference in percentage points between the multiples of three (in percent) and the contribution of multiples from three to all rare events |ζ| > 3 (in percent), and σ_Poisson,%_ = 100[#|ζ| > 3(*m*_3_)]^1/2^/(#|ζ| > 3) is the standard deviation based on Poisson statistics, expressed in percentage points. This is calculated by taking the square root of the number # of rare events |ζ| > 3 from multiples of three *m*_3_, divided by the total number of rare events #|ζ| > 3 (which gives the fraction of rare events from multiples of three), multiplied by 100 to obtain the percentage points.

**Table 3:** Overview of systematic errors in data sets **1**–**5** An × in an entry indicates that traces of this systematic error were found in the data set.

Model	Disorder	Extinction	Significant shift† of ζ	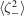 < α^2^ ‡	Overfitting§	Large *wR*(*F* ^2^)	*I*_o_ > *I*_c_	Large aGoF	sinθ/λ dependence¶ of ζ	sinθ/λ dependence†† of ζ^2^	Over-compen-sation‡‡	Under-compen-sation§§	Broken symmetry¶¶ in ζ
**1_uncorr**			×	×		×			×				
**1_corr**						×			×	×	×		
**1_filter**						×			×	×			
**2_uncorr**					×	×			×	×			
**2_corr**					×	×			×	×			
**2_filter**					×	×			×	×			
**3_uncorr**	×		×	×			×		×	×			
**3_corr**	×		×	×			×		×	×	×	×	
**3_filter**	×				×		×		×	×			
**4_uncorr**	×		×	×	×	×	×		×	×			×
**4_corr**	×		×	×	×	×	×		×	×			×
**4_filter**	×		×		×	×	×		×	×			×
**5_uncorr**		×						×	×	×			×
**5_corr**		×						×	×	×			×
**5_filter**		×						×	×	×			×

† Compare with column 5 in Table 1 ▸.

‡ Compare with column 12 in Table 1 ▸.

§ As given by aGoF ≤ 1, compare with column 5 in Table 1 ▸.

¶ As given by χ^2^(ζ, sinθ/λ) > 149, compare with the supporting information.

†† As given by χ^2^(ζ^2^, sinθ/λ) > 149.

‡‡ As shown by histograms showing the 10 or 20% most *negative* residuals with a signicantly larger number of *m*_3_ after correction. Compare with plots in the supporting information.

§§ As shown by histograms showing the 10 or 20% most *positive* residuals with a signicantly larger number of *m*_3_ after correction. Compare with plots in the supporting information.

¶¶ As given by simultaneously showing χ^2^(ζ, *I*_c_) > 149, χ^2^[ζ, σ(*I*_o_)] > 149, χ^2^[ζ, *I*_c_/σ(*I*_o_)] > 149, χ^2^(ζ, sinθ/λ) > 149, compare with the supporting information.

**Table:** 

	**mero-02**	**mero-06**	**nmero1-02**	**nmero1-07**
〈ζ〉/σ(〈ζ〉)	19.84	−0.47	5.98	−4.92
(#ζ_+_ − #ζ_−_)/(*N*_obs_)^1/2^	9.88	2.64	−5.46	−5.67

**Table:** 

	**nmero2-02**	**nmero2-03**	**pmero-02**	**pmero-03**
〈ζ〉/σ(〈ζ〉)	−0.99	−0.53	7.56	−0.78
(#ζ_+_ − #ζ_−_)/(*N*_obs_)^1/2^	−2.37	−0.56	3.55	−0.24

**Table:** 

	**ret1-02**	**ret1-03**	**ret2-02**	**ret2-09**
〈ζ〉/σ(〈ζ〉)	5.87	4.94	20.22	3.69
(#ζ_+_ − #ζ_−_)/(*N*_obs_)^1/2^	3.40	3.81	11.04	4.33

**Table:** The number of positive excess residuals is reduced in all cases by modelling of disorder.

	**Ga-01** †	**Ga-06**	**benz-01** †	**benz-04**
〈ζ〉/σ(〈ζ〉)	6.24	1.77	12.19	−3.03
(#ζ_+_ − #ζ_−_)/(*N*_obs_)^1/2^	5.44	1.52	2.30	−2.52

**Table:** 

	**Ti-01** ‡	**Ti-07**	**Tol-01** §	**Tol-05**
〈ζ〉/σ(〈ζ〉)	23.47	3.43	9.14	9.19
(#ζ_+_ − #ζ_−_)/(*N*_obs_)^1/2^	15.67	1.54	5.14	4.21

† Disorder of two ethyl groups.

‡ Disorder of benzoic acid molecule on a twofold axis.

§ Disorder of a Ti^III^ cation.

¶ Disorder of a toluene solvent molecule about a special position.

## Footnotes

1 Relative correctness to each other is not sufficient, which can easily be seen from a *Gedankenexperiment* in which the σ(*I*_o_) are all too large by a factor of, say, 1.5. This would eliminate all rare events immediately.

2 In aviation it is common practice to examine every single accident in great detail in order to learn more about how this can be avoided. In crystallography one could examine the cases in which deviations from expected values are significant so as to also learn from, and improve, the experiments systematically over the course of time.

3 This may appear counterintuitive at first glance, but when the weighted agreement factor is written in the form *wR*(*F* ^2^) = {〈ζ^2^〉/〈[*I*_o_/σ(*I*_o_)]^2^〉}^1/2^ it is seen that the numerator has an order of magnitude of one, whereas the denominator has an order of magnitude of 10 or 100. Application of a weighting scheme lowers both, but the denominator, the mean squared significance, is lowered faster, as the weighting scheme limits in particular the largest value for the significance, such that the resulting agreement factors tend to increase. A detailed example of this is discussed in the cited literature.

4 For example, reference data sets **2_uncorr** and **2_corr** both show aGoF < 1, whereas **2_filter** shows aGoF = 1.00. As there are, however, remaining systematic errors in data set **2_filter**, it must be concluded that overfitting is also present in reference data set **2_filter** despite aGoF = 1.00.

## References

[bb39] Abou, A., Bamba, F., Marrot, J., Yaya, S. & Coustard, J.-M. (2021). *IUCrData*, **6**, x210674. 10.1107/S241431462100674X PMC9462342 36337322

[bb1] Abrahams, S. C. & Keve, E. T. (1971). *Acta Cryst.* A**27**, 157–165.

[bb45] Castaldi, K. T., Astashkin, A. V., Albert, D. R. & Rajaseelan, E. (2021). *IUCrData*, **6**, x211142. 10.1107/S2414314621011421 PMC9462289 36337466

[bb2] Dittrich, B., Fabbiani, F. P. A., Henn, J., Schmidt, M. U., Macchi, P., Meindl, K. & Spackman, M. A. (2018). *Acta Cryst.* B**74**, 416–426. 10.1107/S2052520618010120 30297547

[bb37] El-Hiti, G. A., Abdel-Wahab, B. F., Yousif, E., Hegazy, A. S. & Kariuki, B. M. (2021). *IUCrData*, **6**, x210318. 10.1107/S2414314621003187 PMC9462327 36339101

[bb30] Ha, K. (2021*a*). *IUCrData*, **6**, x210083. 10.1107/S2414314621000833 PMC9462262 36340471

[bb31] Ha, K. (2021*b*). *IUCrData*, **6**, x210085. 10.1107/S2414314621000857 PMC9462261 36340469

[bb32] Ha, K. (2021*c*). *IUCrData*, **6**, x210093. 10.1107/S2414314621000936 PMC9462264 36340472

[bb33] Ha, K. (2021*d*). *IUCrData*, **6**, x210094. 10.1107/S2414314621000948 PMC9462313 36338856

[bb34] Ha, K. (2021*e*). *IUCrData*, **6**, x210153. 10.1107/S241431462100153X PMC9462318 36338858

[bb3] Hamilton, W. C. (1965). *Acta Cryst.* **18**, 502–510.

[bb4] Henn, J. (2016). *Acta Cryst.* A**72**, 696–703. 10.1107/S2053273316013206 27809209

[bb5] Henn, J. (2019). *Crystallogr. Rev.* **25**, 83–156.

[bb6] Henn, J. & Meindl, K. (2014*a*). *Acta Cryst.* A**70**, 248–256. 10.1107/S2053273314000898 24815974

[bb7] Henn, J. & Meindl, K. (2014*b*). *Acta Cryst.* A**70**, 499–513. 10.1107/S2053273314012984 25176997

[bb8] Henn, J. & Schönleber, A. (2013). *Acta Cryst.* A**69**, 549–558. 10.1107/S0108767313022514 24132216

[bb9] Holton, J. M., Classen, S., Frankel, K. A. & Tainer, J. A. (2014). *FEBS J.* **281**, 4046–4060. 10.1111/febs.12922 PMC4282448 25040949

[bb10] Hooft, R. W. W., Straver, L. H. & Spek, A. L. (2009). *Acta Cryst.* A**65**, 319–321. 10.1107/S0108767309009908 19535853

[bb43] Hu, Q., Wen, B. & Fan, C. (2021). *IUCrData*, **6**, x210988. 10.1107/S2414314621009883 PMC9462374 36338944

[bb11] Ivlev, S. I. & Kraus, F. (2021). *IUCrData*, **6**, x210735. 10.1107/S2414314621007355 PMC9462354 36340658

[bb12] Krause, L., Herbst-Irmer, R. & Stalke, D. (2015). *J. Appl. Cryst.* **48**, 1907–1913. 10.1107/S1600576714022985 PMC4453166 26089746

[bb13] Macchi, P., Bürgi, H.-B., Chimpri, A. S., Hauser, J. & Gál, Z. (2011). *J. Appl. Cryst.* **44**, 763–771.

[bb44] Meenatchi, C. S., Athimoolam, S., Suresh, J., Rubina, S. R., Kumar, R. R. & Bhandari, S. R. (2021). *IUCrData*, **6**, x211195. 10.1107/S2414314621011950 PMC9462290 36337467

[bb14] Meindl, K. & Henn, J. (2010). *Residual Density Analysis.* Heidelberg: Springer.

[bb22] Müller, P. (2006). Editor. *Crystal Structure Refinement: A Crystallographer’s Guide to *SHELXL*.* Oxford University Press/IUCr.

[bb15] Niepötter, B., Herbst-Irmer, R. & Stalke, D. (2015). *J. Appl. Cryst.* **48**, 1485–1497.

[bb16] Ovalle, M. A., Romero, J. A. & Aguirre, G. (2021). *IUCrData*, **6**, x201663. 10.1107/S2414314620016636 PMC9462268 36340467

[bb46] Pacifico, J. & Stoeckli-Evans, H. (2021). *IUCrData*, **6**, x211295. 10.1107/S2414314621012955 PMC9462308 36337591

[bb17] Patel, D. G., Cox, J. M., Bender, B. M. & Benedict, J. B. (2021). *IUCrData*, **6**, x211016. 10.1107/S2414314621010166 PMC9462299 36340984

[bb36] Sathya, U., Nirmal Ram, J. S., Gomathi, S., Ramu, S., Jegan Jennifer, S. & Ibrahim, A. R. (2021). *IUCrData*, **6**, x210379. 10.1107/S2414314621003795 PMC9462331 36339106

[bb18] Sivapriya, S., Priyanka, S., Gopalakrishnan, M., Manikandan, H. & Selvanayagam, S. (2021). *IUCrData*, **6**, x210500. 10.1107/S2414314621005009 PMC9462338 36338267

[bb40] Su, W., Fu, T. & Xu, Z. (2021). *IUCrData*, **6**, x210693. 10.1107/S2414314621006933 PMC9462358 36340661

[bb41] Sung, J. (2021). *IUCrData*, **6**, x210950. 10.1107/S2414314621009500 PMC9462370 36338949

[bb35] Vinotha, G., Sundar, T. V. & Sharmila, N. (2021). *IUCrData*, **6**, x210210. 10.1107/S2414314621002108 PMC9462310 36338860

[bb19] Williams, A. E., Thompson, A. L. & Watkin, D. J. (2019). *Acta Cryst.* B**75**, 657–673. 10.1107/S2052520619006681 32830722

[bb42] Yaffa, L., Pouye, S. F., Ndoye, D., Diallo, W., Diop, M., Sidibe, M. & Diop, C. A. K. (2021). *IUCrData*, **6**, x210982. 10.1107/S2414314621009822 PMC9462368 36338947

[bb38] Yang, X. & Long, S. (2021). *IUCrData*, **6**, x210539. 10.1107/S2414314621005393 PMC9462340 36338272

[bb20] Yoo, M. & Koh, D. (2021*a*). *IUCrData*, **6**, x210096. 10.1107/S2414314621000961 PMC9462267 36340475

[bb21] Yoo, M. & Koh, D. (2021*b*). *IUCrData*, **6**, x210590. 10.1107/S2414314621005903 PMC9462352 36337328

