# Supplementary material for: Progress in detection of and correction for low-energy contamination
Source: J Appl Crystallogr. 2023 Jul 25;56(Pt 4):1200–20. doi: 10.1107/S1600576723004764 (PMC10405589; doi:10.1107/S1600576723004764)
Supplement: Supplementary file 1 [file j-56-01200-sup1.pdf]

JOURNAL OF
APPLIED
CRYSTALLOGRAPHY

Volume 56 (2023)

Supporting information for article:

Progress in detection of and correction for low-energy contamination

Slawomir Domagala, Petrick Nourd, Kay Diederichs and Julian Henn

The supplementary material contains:

Table S1: The plots of 2-lambda, 3-lambda and 6-lambda signals for data sets 1-5 (uncorr, corr, filter) together with the corresponding histograms for 3-lambda contamination.

Table S2: Characteristic plots including the "balance sheet plot”, normal probability plot, a plot of moving averages of the weighted residuals sorted in ascending order of resolution, BayCoN plot ($\zeta, \sin\theta/\lambda$) and scatter plot $I_o\ vs.\ I_c$ for data sets 1-5 (uncorr, corr, filter)

Table S3: The χ^2- values of the BayCoN plots for data sets 1-5 (uncorr, corr, filter) (Table 3). χ^2-values larger than 149 disprove uniformity of the plots, *i.e.* they establish a systematic (non-linear) connection between the residuals (or squared residuals) and the corresponding property, *e.g.* resolution.

Table S4: The fractal dimension plots for disordered structures published by Peter Müller in BOOK PM

Table S5: A list of data sets from all data sets of IUCrData published in 2021, for which the ratio of observed contributions to rare events from multiples of three to expected contributions of multiples of three to rare events is two or larger.

Table S1: Histograms of multiples of 3 and of 2 in bins of (squared) weighted residuals

A	B	C	D
		$m_3(\zeta,5)$	$m_3(\zeta,10)$
1_uncorr	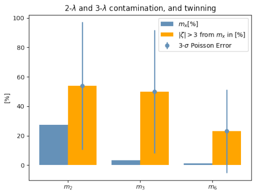	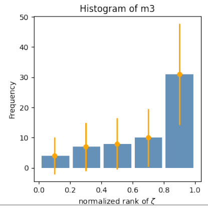	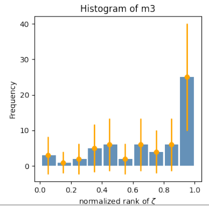
1_corr	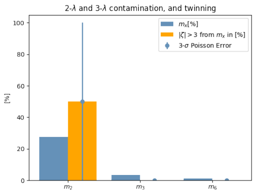	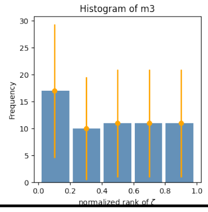	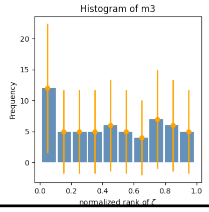
1_filter	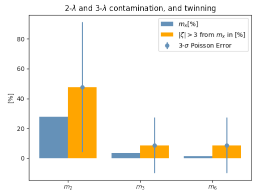	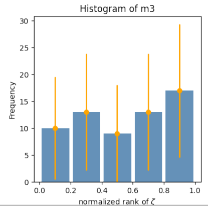	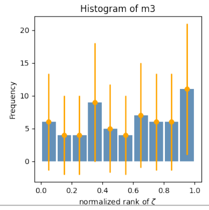
2_unorr	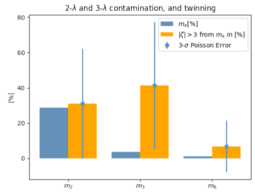	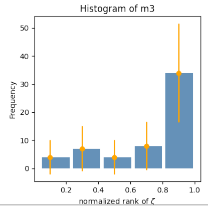	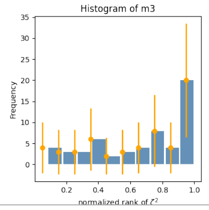
2_corr	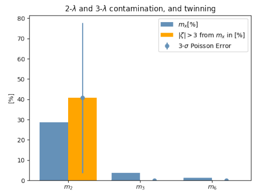	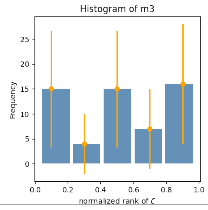	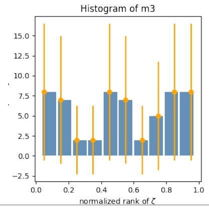
2_filter	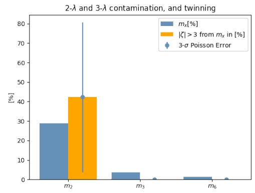	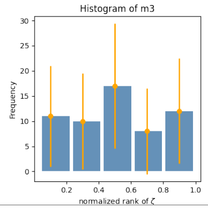	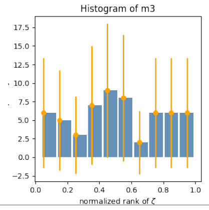
3_uncorr	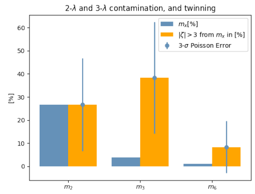	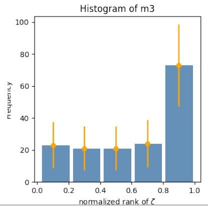	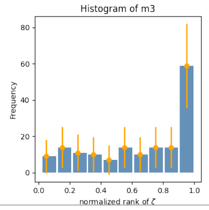
3_corr	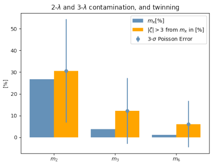	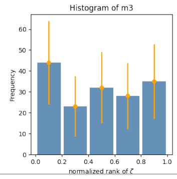	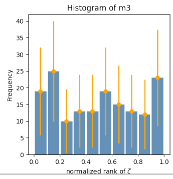

A	B	C	D
		$m_3(\zeta,5)$	$m_3(\zeta,10)$
3_filter	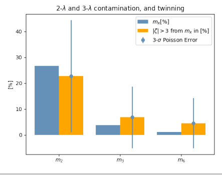	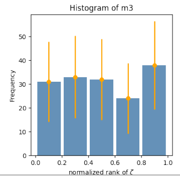	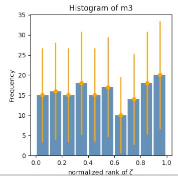
4_uncorr	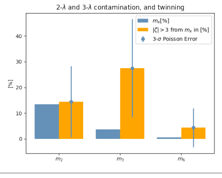	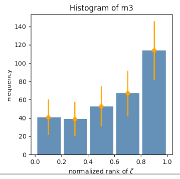	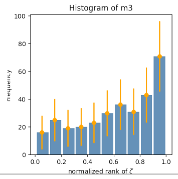
4_corr	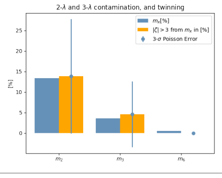	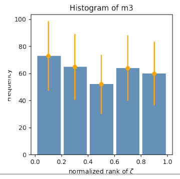	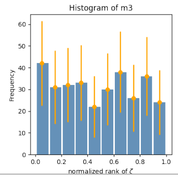
4_filter	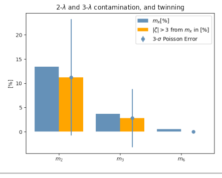	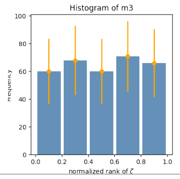	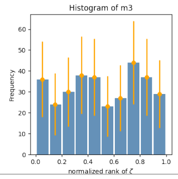
5_uncorr	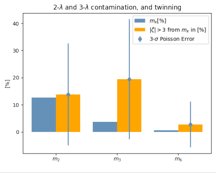	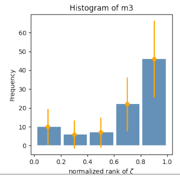	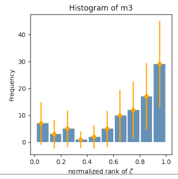
5_corr	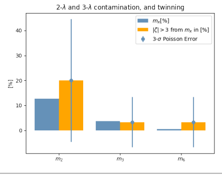	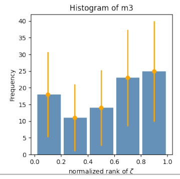	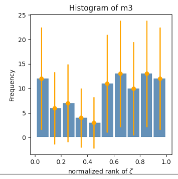
5_filter	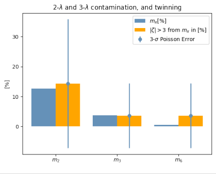	Twin? 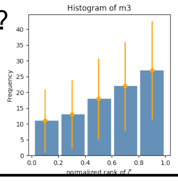	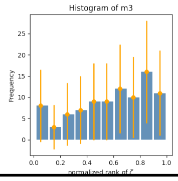

Histograms of multiples of 3 (columns **C,D**) in bins of weighted residuals with different number of bins (5, columns **C**,**G**,**E**,**I;** 10, columns **D**, **F**, **H**, **J;** 15, column **K**)

Table 2: Characteristic plots of all data sets

A	B	C	D	E	F
	Fraction of positive and negative residuals, mean of positive and of absolute negative residuals and squared residuals	**Normal probability plot**	**Moving averages of** ζ **in order of** $\sin\theta/\lambda$	**BayCoN** $(\zeta, \sin\theta/\lambda)$	**Scatter plot** I_o **vs.** I_c
1_uncorr	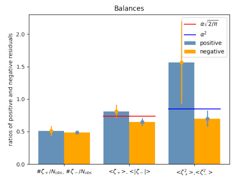	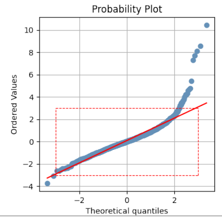	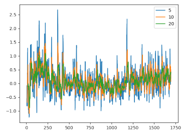	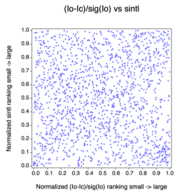	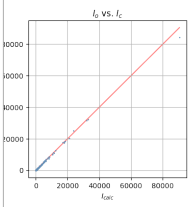
1_corr	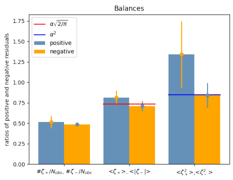	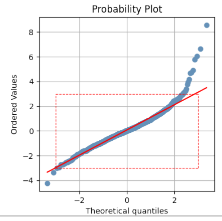	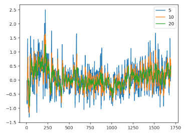	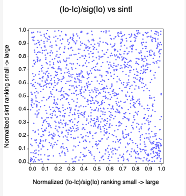	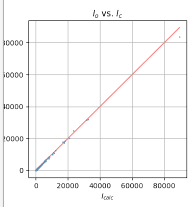
1_filter	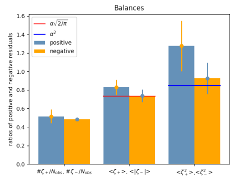	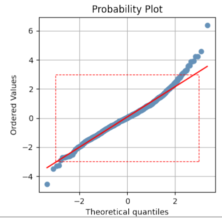	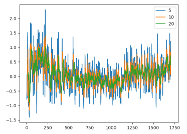	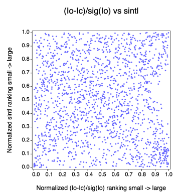	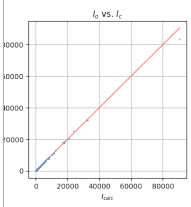
2_unorr	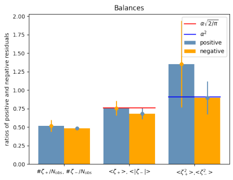	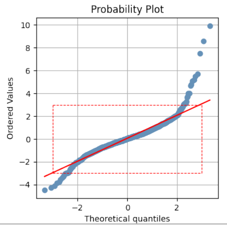	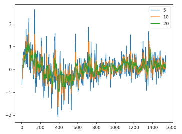	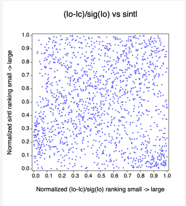	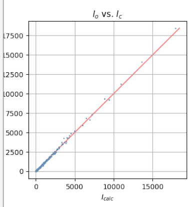
2_corr	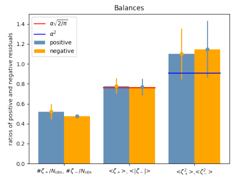	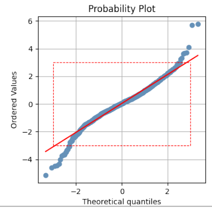	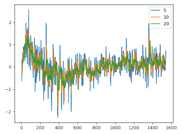	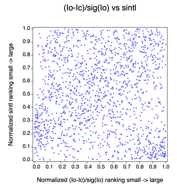	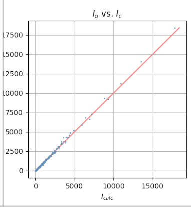
2_filter	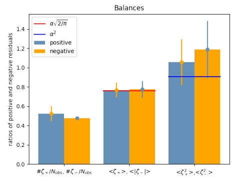	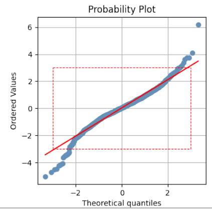	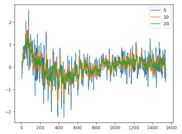	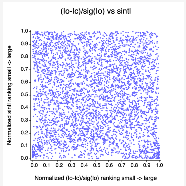	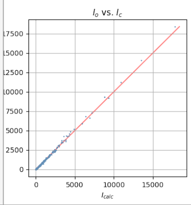

<table>
<tr><th>A</th><th>B</th><th>C</th><th>D</th><th>E</th><th>F</th></tr>
<tr><td></td><td>Fraction of positive and negative residuals, mean of positive and of absolute negative residuals and squared residuals</td><td>Normal probability plot</td><td>Moving averages of ζ in order of $\sin\theta/\lambda$</td><td>BayCoN $(\zeta, \sin\theta/\lambda)$</td><td>Scatter plot I_o vs. I_c</td></tr>
<tr><td>3_uncorr</td><td>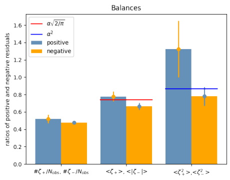</td><td>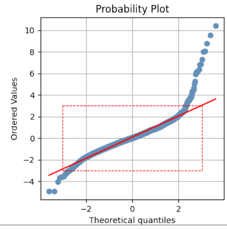</td><td>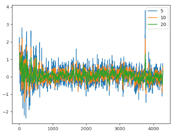</td><td>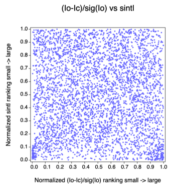</td><td>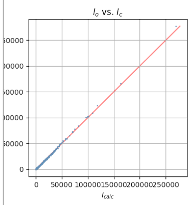</td></tr>
<tr><td>3_corr</td><td>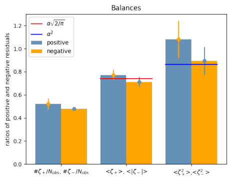</td><td>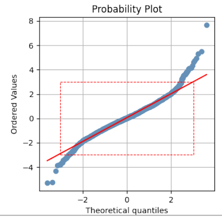</td><td>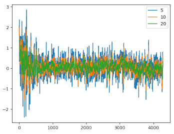</td><td>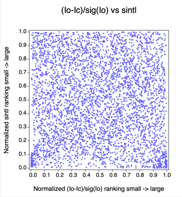</td><td>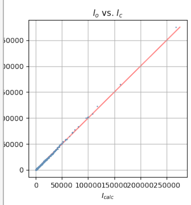</td></tr>
<tr><td>3_filter</td><td>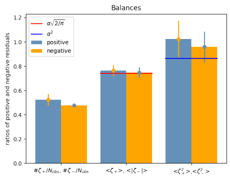</td><td>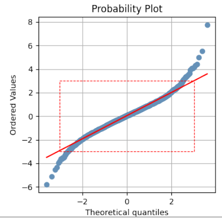</td><td>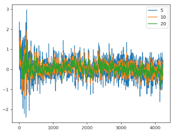</td><td>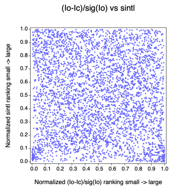</td><td>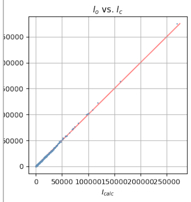</td></tr>
<tr><td>4_uncorr</td><td>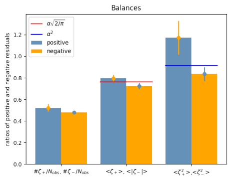</td><td>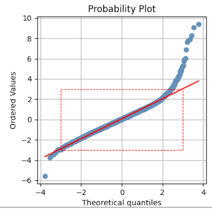</td><td>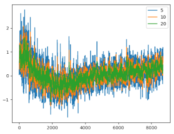</td><td>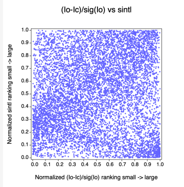</td><td>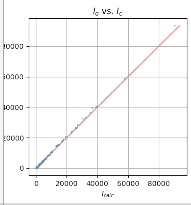</td></tr>
<tr><td>4_corr</td><td>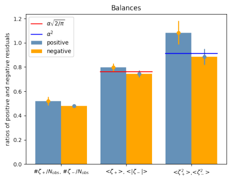</td><td>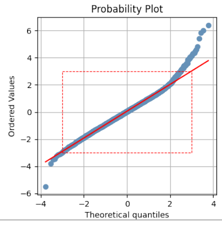</td><td>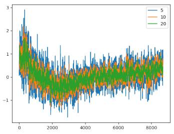</td><td>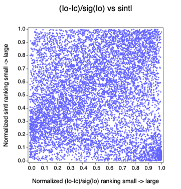</td><td>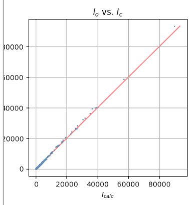</td></tr>
<tr><td>4_filter</td><td>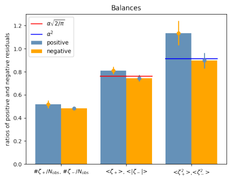</td><td>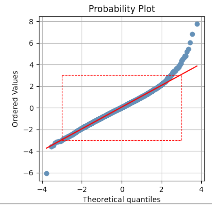</td><td>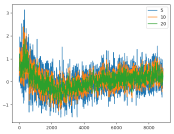</td><td>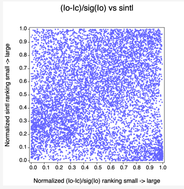</td><td>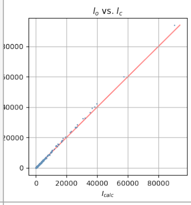</td></tr>
<tr><td>5_uncorr</td><td>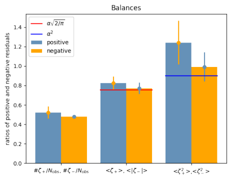</td><td>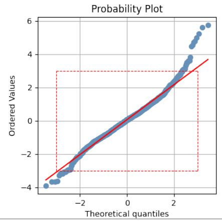</td><td>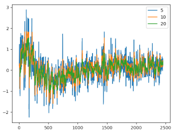</td><td>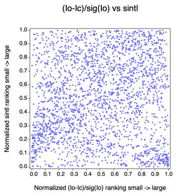</td><td>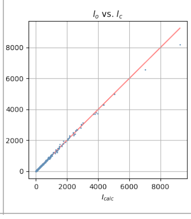</td></tr>
</table>

A	B	C	D	E	F
	Fraction of positive and negative residuals, mean of positive and of absolute negative residuals and squared residuals	**Normal probability plot**	**Moving averages of** ζ **in order of** $\sin\theta/\lambda$	**BayCoN** $(\zeta, \sin\theta/\lambda)$	**Scatter plot** I_o **vs.** I_c
5_corr	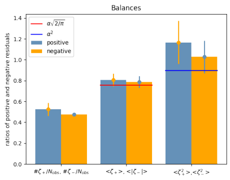	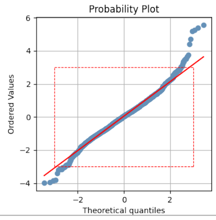	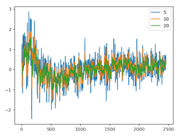	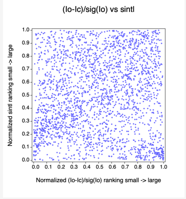	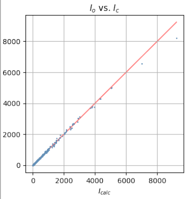
5_filter	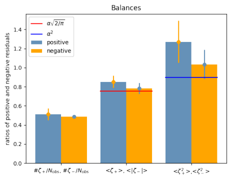	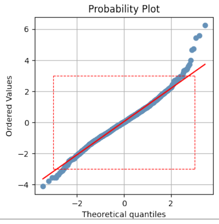	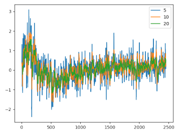	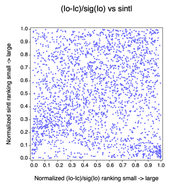	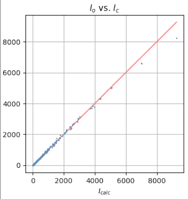

Column **B** shows (left) the fraction of positive (blue) and of negative (orange) residuals, (middle) the mean value of positive residuals (blue) and the absolute mean value of negative residuals (orange), and (right) the mean value of the squared positive residuals (blue) and of the squared negative residuals (orange). Column **C** shows the normal probability plot (npp). In column **D**, the moving averages of the weighted residuals sorted in ascenending order of the resolution are plotted. Column **D** show the BayCoN plots of the residuals and the resolution and in column **D**, the scatter plots of observed *versus* calculated intensities is depicted.

Table S3: chi^2 values of BayCoN plots

	A	B	C	D	E	F	G	H
	(ζ, I_c)	$(\zeta, \sigma(I_o))$	$(\zeta, I_c/\sigma(I_o))$	$(\zeta, \sin\theta/\lambda)$	(ζ^2, I_c)	$(\zeta^2, \sigma(I_o))$	$(\zeta^2, I_c/\sigma(I_o))$	$(\zeta^2, \sin\theta/\lambda)$
1_uncorr	97,64	88,80	106,36	**154,44**	76,19	74,54	72,53	143,48
1_corr	91,63	97,40	97,64	**191,33**	79,49	74,77	81,49	**169,41**
1_filter	102,31	94,82	107,70	**195,23**	90,36	85,79	82,87	**176,71**
2_uncorr	135,22	134,71	136,39	**283,90**	94,65	99,45	97,38	**203,53**
2_corr	131,21	136,91	133,15	**169,31**	88,04	87,40	91,80	**189,40**
2_filter	95,71	90,40	95,84	**276,94**	89,89	79,14	99,60	**219,72**
3_uncorr	149,76	130,56	**170,87**	**305,48**	98,99	76,57	117,11	**408,80**
3_corr	136,96	116,41	**160,74**	**313,19**	88,25	82,22	83,81	**362,09**
3_filter	130,26	107,40	**191,12**	**323,37**	130,26	107,40	**191,12**	**315,71**
4_uncorr	**238,64**	**258,56**	**332,27**	**1208,02**	139,01	120,19	139,82	**615,85**
4_corr	**224,06**	**251,85**	**299,79**	**1265,37**	138,60	108,99	**151,3**	**637,15**
4_filter	**287,03**	**225,36**	**386,38**	**1023,96**	127,68	99,51	148,71	**528,59**
5_uncorr	**182,93**	**186,35**	**200,51**	**451,90**	95,47	107,03	109,88	**287,49**
5_corr	**184,64**	**203,12**	**192,86**	**455,40**	116,96	117,29	118,59	**291,39**
5_filter	**207,38**	**212,35**	**205,17**	**446,76**	114,67	116,79	120,46	**279,78**
Average	159,69	155,67	187,79	470,97	104,57	95,81	113,76	321,94

χ^2values of weighted residuals (columns **A**-**D**) and of squared weighted residuals (columns **E**-**H**) for corresponding BayCoN plots. Values larger than 149 are printed in bold and disprove uniformity. The largest value is underlined for each category. It always appears for the resolution.

Table S4: fractal dimension plots for structures with disorder being modelled and not being modelled

Ga-01	Ga-06	Benz-01	Benz-03
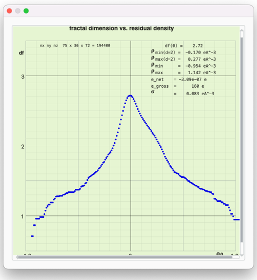	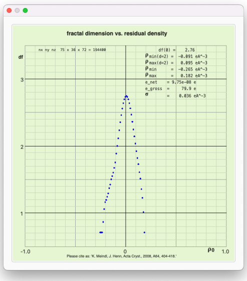	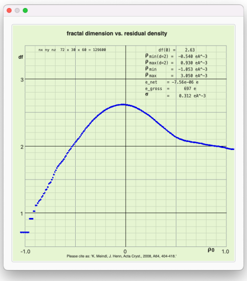	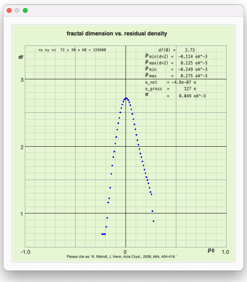

Ti-01	Ti-07	Tol-01	Tol-05
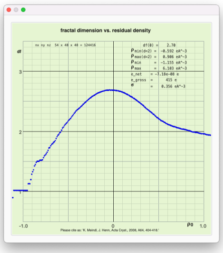	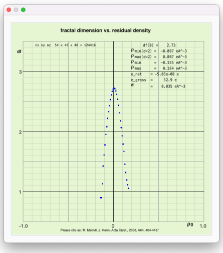	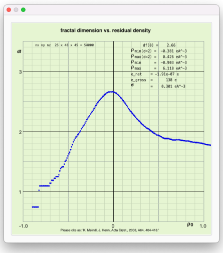	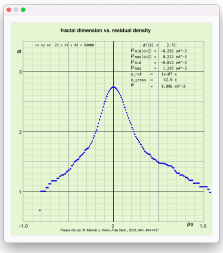

Table S5: List of 23 data sets from iucrdata 2021 with ratio m3 larger than two and significance of m3 signal

	Internet reference	Reference	Ratio of found and expected fraction of m3	Significance of m3 signal			
1	https://doi.org/10.1107/S2414314621000961	Yoo, M. & Koh, D. (2021). IUCrData 6.	8.3667	2.4904	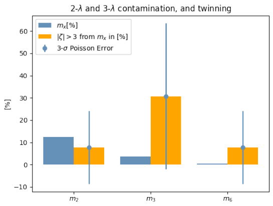	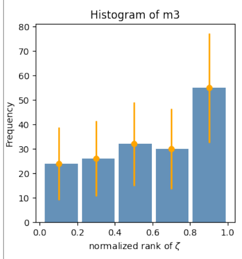	Mo
4	https://doi.org/10.1107/S2414314620016636	Ovalle, M. A., Romero, J. A. & Aguirre, G. (2021). IUCrData 6.	2.0734	1.1576	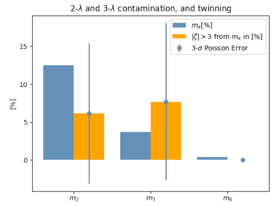	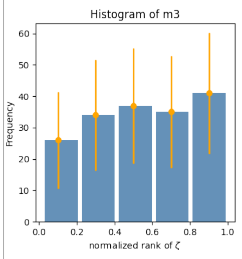	Cu
5	https://doi.org/10.1107/S2414314621000936	Ha, K. (2021). IUCrData 6.	2.5540	1.0539	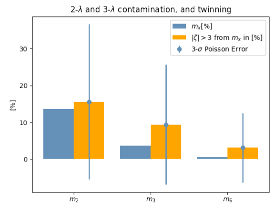	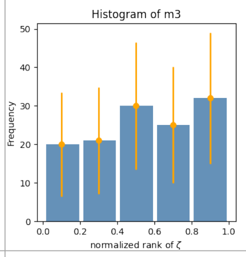	Mo
6	https://doi.org/10.1107/S2414314621000857	Ha, K. (2021). IUCrData 6.	2.7551	1.2741	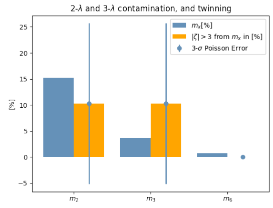	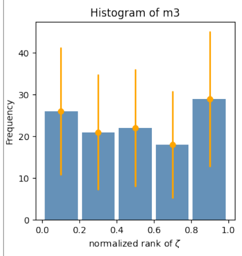	Mo
7	https://doi.org/10.1107/S2414314621000833	Ha, K. (2021). IUCrData 6.	2.8865	0.6536	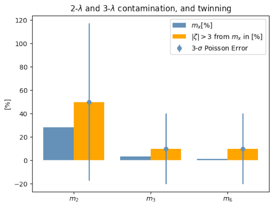	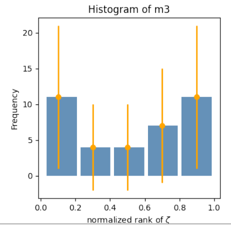	Mo
11	https://doi.org/10.1107/S2414314621002108	Vinotha, G., Sundar, T. V. & Sharmila, N. (2021). IUCrData 6.	2.7377	1.2695	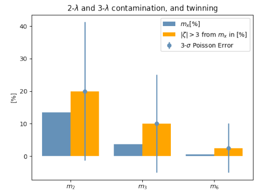	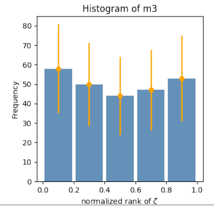	Mo

	Internet reference	Reference	Ratio of found and expected fraction of m3	Significance of m3 signal			
19	https://doi.org/10.1107/S241431462100153X	Ha, K. (2021). IUCrData 6.	2.0536	1.1472	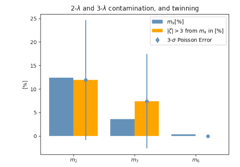	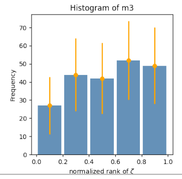	Mo
20	https://doi.org/10.1107/S2414314621000948	Ha, K. (2021). IUCrData 6.	2.6511	1.0787	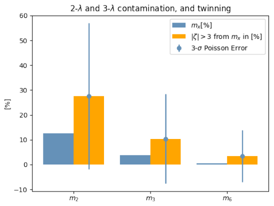	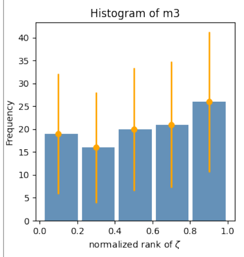	Mo
29	https://doi.org/10.1107/S2414314621003795	Sathya, U., Nirmal Ram, J. S., Gomathi, S., Ramu, S., Jegan Jennifer, S. & Ibrahim, A. R. (2021). IUCrData 6.	2.0166	0.7129	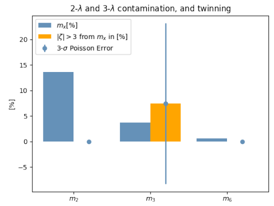	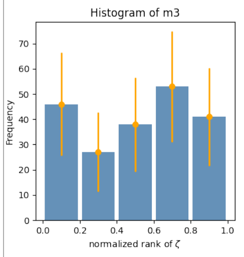	Mo
31	https://doi.org/10.1107/S2414314621003187	El-Hiti, G. A., Abdel-Wahab, B. F., Yousif, E., Hegazy, A. S. & Kariuki, B. M. (2021). IUCrData 6.	4.6797	0.7863	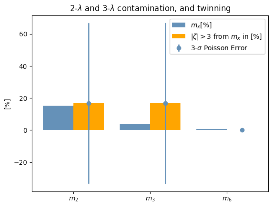	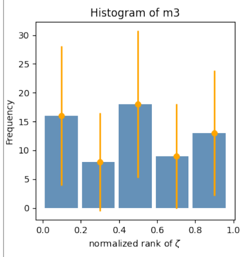	Cu
36	https://doi.org/10.1107/S2414314621005393	Yang, X. & Long, S. (2021). IUCrData 6.	2.0979	0.9064	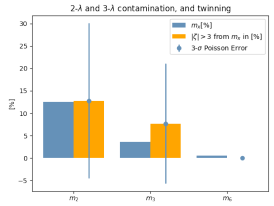	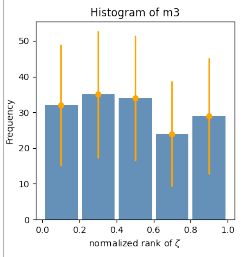	Mo
37	https://doi.org/10.1107/S2414314621005009	Sivapriya, S., Priyanka, S., Gopalakrishnan, M., Manikandan, H. & Selvanayagam, S. (2021). IUCrData 6.	6.4529	2.6722	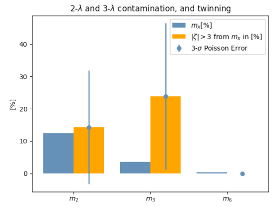	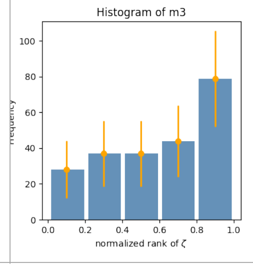	Mo

	Internet reference	Reference	Ratio of found and expected fraction of m3	Significance of m3 signal			
43	https://doi.org/10.1107/S241431462100674X	Abou, A., Bamba, F., Marrot, J., Yaya, S. & Coustard, J.-M. (2021). IUCrData 6.	2.4307	0.8324	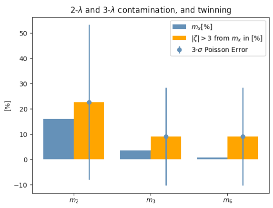	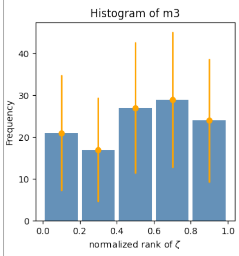	Mo
45	https://doi.org/10.1107/S2414314621005903	Yoo, M. & Koh, D. (2021). IUCrData 6.	12.5172	3.3175	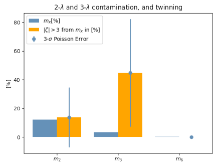	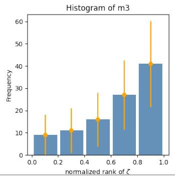	Mo
57	https://doi.org/10.1107/S2414314621006933	Su, W., Fu, T. & Xu, Z. (2021). IUCrData 6.	2.2066	1.2227	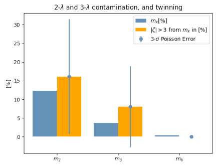	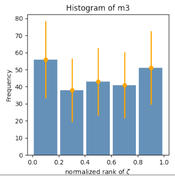	Mo
60	https://doi.org/10.1107/S2414314621007355	Ivlev, S. I. & Kraus, F. (2021). IUCrData 6.	3.5865	0.7212	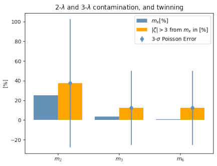	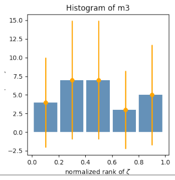	Cu
69	https://doi.org/10.1107/S2414314621009500	Sung, J. (2021). IUCrData 6.	6.0493	2.8915	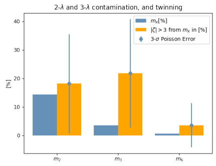	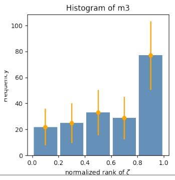	Mo
72	https://doi.org/10.1107/S2414314621009822	Yaffa, L., Pouye, S. F., Ndoye, D., Diallo, W., Diop, M., Sidibe, M. & Diop, C. A. K. (2021). IUCrData 6.	2.3151	0.9839	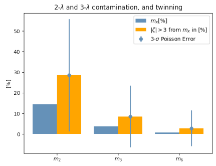	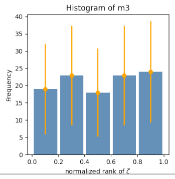	Mo
73	https://doi.org/10.1107/S2414314621009883	Hu, Q., Wen, B. & Fan, C. (2021). IUCrData 6.	2.0062	0.7093	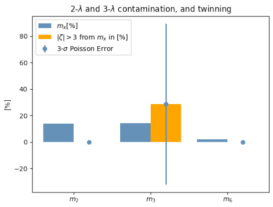	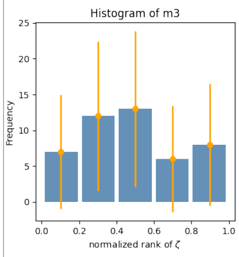	Mo

	Internet reference	Reference	Ratio of found and expected fraction of m3	Significance of m3 signal			
79	https://doi.org/10.1107/S2414314621010166	Patel, D. G., Cox, J. M., Bender, B. M. & Benedict, J. B. (2021). IUCrData 6.	5.9120	2.8782	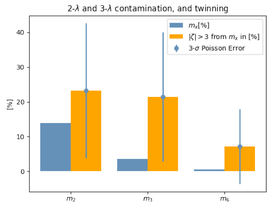	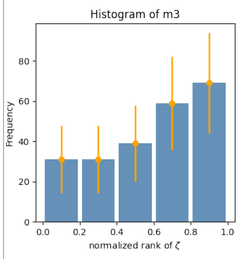	Mo
85	https://doi.org/10.1107/S2414314621011950	Meenatchi, C. S., Athimoolam, S., Suresh, J., Rubina, S. R., Kumar, R. R. & Bhandari, S. R. (2021). IUCrData 6.	2.9256	0.9308	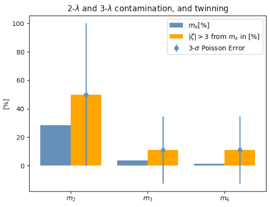	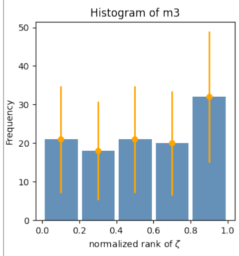	Mo
89	https://doi.org/10.1107/S2414314621011421	Castaldi, K. T., Astashkin, A. V., Albert, D. R. & Rajaseelan, E. (2021). IUCrData 6.	2.2430	1.1083	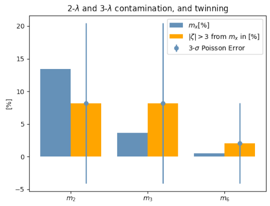	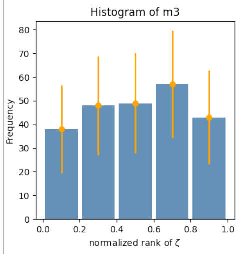	Mo
94	https://doi.org/10.1107/S2414314621012955	Pacifico, J. & Stoeckli-Evans, H. (2021). IUCrData 6.	2.1913	1.0873	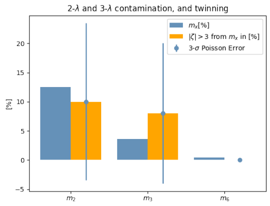	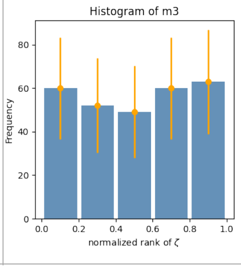	Mo
